# Gamma-Glutamyl Transferase and Cognitive Outcomes: A Cross-Species Analysis and Hepatic Transcriptomic Signature

**DOI:** 10.3390/life16101735

**Published:** 2026-10-16

**Authors:** Rongjiang Zhang, Tianhao Yu, Kai Hao, Hui Lu, Wenting Zhang, Ying Yu, Lingxiao Chen, Xiaorui Cheng

**Affiliations:** 1 Innovative Institute of Chinese Medicine and Pharmacy, Shandong University of Traditional Chinese Medicine, Jinan 250355, China; 2 School of Mathematics and Statistics, Qilu University of Technology (Shandong Academy of Sciences), Jinan 250355, China; 3 Shandong Artificial Intelligence Institute, Qilu University of Technology (Shandong Academy of Sciences), Jinan 250355, China

**Keywords:** liver-brain axis, cognitive impairment, nonlinear association, gamma-glutamyl transferase, virtual gene knockout, immunoglobulin, single-cell transcriptomics

## Abstract

Cognitive impairment is a major public health concern, and the liver–brain axis has attracted growing interest. Gamma-glutamyl transferase (GGT) is a serum marker of liver function, but its association with cognitive outcomes remains uncertain. We conducted a cross-species analysis. In *5xFAD* mice (*n* = 52), we examined serum liver markers and spatial cognition using the Morris water maze. In NHANES 2011–2014 (*n* = 3181, aged ≥60 years), we evaluated liver markers and low cognitive performance (LCP) using multivariable logistic regression. We also performed virtual hepatic *GGT1* knockout in a human liver single-cell atlas and integrated GTEx liver co-expression, transcription factor enrichment, and WGCNA analyses. In mice, GGT–cognition associations largely attenuated after strain adjustment, but a positive association with platform crossings emerged (r = 0.321, *p* = 0.02). In NHANES, serum GGT was inversely associated with LCP (Q4 vs. Q1 OR = 0.54, *p* < 0.001), with a nonlinear association and an inflection point near 19 U/L. Virtual hepatic *GGT1* knockout identified eight immunoglobulin-related genes associated with *SPI1* and oxidative stress-related pathways. These parallel findings are hypothesis-generating. A testable hypothesis is that lower hepatic *GGT1* activity or serum GGT is associated with upregulated cholangiocyte immunoglobulin-related programs and poorer cognition.

## 1. Introduction

Cognitive impairment is an important public health concern and is a major feature of Alzheimer’s disease (AD) and related dementias. Although research has traditionally focused on central nervous system pathology, increasing amounts of evidence indicates that systemic metabolic, inflammatory, and vascular processes may also influence brain function [1,2,3,4,5]. The liver is a major metabolic and immune organ and may participate in brain health through circulating metabolites, proteins, inflammatory mediators, and redox-related pathways [6,7,8]. This has motivated growing interest in the liver–brain axis as a framework for studying systemic contributions to cognitive function.

Routine serum liver markers—including aspartate aminotransferase (AST), alanine aminotransferase (ALT), alkaline phosphatase (ALP), gamma-glutamyl transferase (GGT), and total protein (TP)—provide readily available measures of hepatic and systemic physiology. Their relationships with cognitive outcomes may reflect multiple biological and non-biological processes, including metabolic dysfunction, inflammation, nutritional status, and oxidative stress [8,9,10]. GGT is involved in extracellular glutathione metabolism and is therefore biologically relevant to redox homeostasis [11,12]. However, serum GGT activity is a clinical biomarker and should not be assumed to be equivalent to hepatic *GGT1* expression or activity. The molecular relationships connecting serum GGT, hepatic *GGT1*, and cognitive outcomes remain uncertain [13,14].

Previous epidemiological studies have reported heterogeneous associations between liver-related biomarkers and cognitive outcomes [15]. Differences in study populations, cognitive phenotypes, exposure definitions, and adjustment strategies may contribute to these inconsistencies [16,17]. A cross-species analysis combining animal behavioral data, population-level epidemiology, genetic analyses, and transcriptomic observations may help to identify reproducible associations and generate testable mechanistic hypotheses, while keeping causal and mechanistic interpretations appropriately separated.

To address this question, we designed a multi-level, cross-species study using three complementary approaches. First, we examined associations between serum liver markers and spatial cognitive performance in the 5xFAD mouse model using the Morris water maze. Second, we evaluated associations between the same markers and low cognitive performance (LCP) in NHANES. Third, we applied virtual hepatic *GGT1* knockout in a human liver single-cell atlas and integrated transcription factor enrichment, GTEx co-expression, mediation, and WGCNA analyses to nominate candidate molecular programs. The overall aim was to characterize cross-species associations and generate hypotheses regarding hepatic molecular pathways that may be relevant to cognition, rather than to establish a causal therapeutic mechanism.

## 2. Materials and Methods

### 2.1. Animal Experiments

#### 2.1.1. Animals and Serum Biochemistry

A total of 52 mice from Jackson Laboratory were used. This cohort included 26 *5xFAD* mice and 26 wild-type (*WT*) mice. Among the *5xFAD* mice, there were 15 females and 11 males, while the *WT* group consisted of 19 females and 7 males. The average body weight of the mice was 25.6 g, and the average age was 15.2 months. *5xFAD* mice express five familial AD mutations (*APP K670N*/*M671L I716V V717I* and *PSEN1 M146L L286V*) leading to rapid cerebral Aβ deposition [18]. Mice were housed under specific pathogen-free conditions at 22–24 °C with ad libitum access to standard chow and sterile water. All procedures were approved by the Animal Care and Use Committee of Shandong University of Traditional Chinese Medicine (Approval No. SDUTCM20251010002). Blood was collected via the orbital sinus prior to behavioral testing. Serum was separated by centrifugation at 3100 rpm (approximately 1290× *g*; rotor radius 12 cm) for 10 min at 4 °C, then an automated biochemical analyzer (BS−360S, jinan, China) was used to test AST, ALT, ALP, GGT, and TP. The BS−360S assay’s LoD/LoQ for GGT was not available at the time of analysis. Because mouse serum GGT concentrations (approximately 2–4 U/L) are close to the lower limit of quantification of many clinical analyzers, the mouse GGT data should be interpreted with caution.

#### 2.1.2. Morris Water Maze

Spatial learning and cognition were assessed using the Morris water maze (MWM) as previously described [19]. Briefly, the apparatus consisted of a circular pool filled with opaque water, with a hidden escape platform fixed in the third quadrant. During the 4-day acquisition phase, mice participated in four trials per day from randomized entry points (60 s maximum per trial, Table S1). Escape latency was recorded, and the area under the curve (AUC) for each training day was calculated as a composite learning metric. On day 5, a 60 s probe trial was conducted with the platform removed. Outcome measures included (1) escape latency to the former platform location, (2) number of platform crossings, (3) time spent in the target quadrant, and (4) distance swum in the target quadrant. A passing threshold (30 s mean escape latency) was used to determine readiness for probe testing; all *WT* mice reached this criterion by day 4. Ten of 26 *5xFAD* mice did not reach the 30 s criterion by day 4. These mice were nevertheless included in the probe trial because the probe trial was conducted at a fixed timepoint for the whole cohort rather than being conditional on individual performance. Swim speed was not recorded, and a visible-platform test was not performed. Therefore, the MWM results should be interpreted as indicative of overall spatial learning and memory performance, which may include motor, visual, or motivational components, rather than as a pure measure of spatial cognition.

#### 2.1.3. Liver Histopathology

After behavioral analysis, three mice from each of the two strains at 4.9, 14.9, 16.5, and 19.5 months of age were selected for histopathological examination of liver tissue. Histopathological features (hepatocyte edema, inflammatory infiltration, necrosis, and fibrosis) were graded semi-quantitatively (0–4 scale) according to INHAND guidelines (Table S2). Histopathological scoring was performed by investigators who were unaware of genotype and behavioral results. The histology cohort was the same as the behavioral cohort.

#### 2.1.4. Statistical Analysis of Mouse Data

Group differences in MWM performance were assessed using two-way ANOVA with strain and sex as between-subject factors and age as a covariate. Age-related trajectories were analyzed separately using linear regression models including age, strain, and an age × strain interaction term. Associations between serum liver markers and MWM outcomes were evaluated using Pearson partial correlations across three hierarchical models: Model 1 (unadjusted), Model 2 (adjusted for age and sex), and Model 3 (adjusted for age, sex, and strain). Statistical significance was set at *p* < 0.05.

### 2.2. Population-Based Analysis

#### 2.2.1. Study Population

The human data came from the 2011–2014 National Health and Nutrition Examination Survey (NHANES). Out of the initial 17,479 participants, we excluded people under 60 years old (*n* = 9241), those who were missing blood test data (*n* = 2472), participants missing cognitive test data (*n* = 1976), and those missing demographic data (*n* = 609). The final analysis included 3181 participants (Figure S1). The NHANES study protocol was approved by the National Center for Health Statistics (NCHS) Ethics Review Committee, and all participants gave written informed consent. To evaluate selection bias, we compared demographic and clinical characteristics of the included participants (*n* = 3181) with those who were excluded due to missing blood data (*n* = 2472) or missing cognitive data (*n* = 1976) using design-based tests. The results are presented in Table S3.

#### 2.2.2. Cognitive Assessment

Cognitive function was assessed using three standardized neuropsychological tests from the NHANES cognitive battery: (1) the CERAD Word Learning and Recall Test (immediate and delayed memory) [20], (2) the Animal Fluency Test (semantic memory and executive function), and (3) the Digit Symbol Substitution Test (processing speed and working memory) [21]. Standardized scores were combined into a composite cognitive score. Participants whose composite score fell below 25% of the theoretical maximum attainable score were classified as having LCP, consistent with methods previously published in the literature [22,23,24]. We emphasize that this is an operational, threshold-based research classification. It is not a clinical diagnosis; the classification does not correspond to Alzheimer’s disease, dementia, or mild cognitive impairment as defined by any diagnostic criteria.

#### 2.2.3. Liver Function Markers and Covariates

Serum AST, ALT, ALP, GGT, and TP were measured using standardized enzymatic assays. Each marker was categorized into quartiles (Q1–Q4) for logistic regression, with Q1 as the reference. Covariates were selected using a directed acyclic graph (DAG) to identify variables that were considered potential common causes of GGT and the cognitive phenotype (Figure S2). The covariates included age, gender, education, marital, BMI was categorized using standard WHO thresholds: underweight (<18.5 kg/m^2^), normal weight (18.5–24.9 kg/m^2^), overweight (25.0–29.9 kg/m^2^), and obesity (≥30.0 kg/m^2^). Alcohol consumption was categorized using sex-specific average daily drinks: none (0 drinks/day); mild (>0 to ≤2 drinks/day for men and >0 to ≤1 drink/day for women); heavy (>2 drinks/day for men and >1 drink/day for women). HDL cholesterol was analyzed as a continuous variable (mg/dL). Sleep duration was analyzed as a continuous variable. A sensitivity analysis restricted to the fasting subsample, using WTSAF2YR and additionally adjusting for LDL, was performed to evaluate the robustness of the findings.

#### 2.2.4. Statistical Analysis of Human Data

All analyses accounted for the complex survey design of NHANES using appropriate sampling weights, strata, and primary sampling units. All descriptive and inferential analyses accounted for the NHANES complex survey design. Baseline characteristics between the LCP and NC groups were compared using design-based tests: Rao-Scott chi-square tests for categorical variables and survey-weighted *t*-tests for continuous variables. The 2011–2014 NHANES cycles were combined, and 4-year MEC examination weights were constructed as WTMEC2YR/2, as recommended by NHANES analytic guidelines. Strata (SDMVSTRA) and primary sampling units (SDMVPSUs) were incorporated in all models. Quartile cut-points for serum liver markers (AST, ALT, ALP, GGT, TP) in U/L are reported in Table S4. Descriptive statistics are presented for sample characterization only. They are unadjusted and are not directly comparable to the multivariable quartile-based ORs, which estimate the odds of LCP relative to the lowest exposure quartile after covariate adjustment. Multivariable logistic regression [25] was used to estimate odds ratios (ORs) and 95% confidence intervals (CIs) for the association between liver marker quartiles and LCP. The primary fully adjusted model (Model 3) included age, gender, education level, marital status, BMI, alcohol consumption, HDL (continuous, mg/dL), and sleep duration. To assess the stability of the regression estimates, multicollinearity was evaluated using variance inflation factors (VIFs) or generalized VIFs, as appropriate for the model parameterization. LDL was not included in the primary full-sample model because it is available only in the morning fasting subsample; its influence was evaluated in a fasting-subsample sensitivity analysis using WTSAF2YR. Primary analyses used MEC examination weights (WTMEC2YR) appropriate for the full sample. Models 1 and 2 also used WTMEC2YR. Restricted cubic splines (RCSs) [26] with three knots were used to evaluate potential nonlinearity for GGT. Subgroup analyses [27] were stratified by age (≤65 vs. >65 years), gender, education, marital, alcohol consumption, BMI, and sleep duration; interaction terms were tested using product terms in the regression models. GGT was categorized using clinically meaningful cut-points: low (<10 U/L), normal (10–60 U/L, reference), and high (≥60 U/L) [28,29]. These cut-points were chosen because they are commonly used in clinical practice, facilitate interpretation of extreme values.

#### 2.2.5. Sensitivity Analysis of Human Data

To address potential residual confounding, additional sensitivity analyses were prespecified using the NHANES 2011–2014 complex survey design. The primary multivariable model (Model 3) was sequentially expanded. As shown in Table S5 Model 4 additionally included race/ethnicity, poverty–income ratio, smoking status, diabetes, hypertension, and cardiovascular disease. Model 5 further included depressive symptoms assessed using the PHQ−9, estimated glomerular filtration rate (eGFR), and physical activity. Model 6 also included anticonvulsant medication use because selected anticonvulsant agents can influence serum GGT concentrations and may be associated with neurological conditions.

Race/ethnicity was categorized according to NHANES demographic classifications. The poverty–income ratio was analyzed as a socioeconomic indicator. Smoking status was categorized as never, former, or current smoker. Diabetes, hypertension, and cardiovascular disease were defined using the available NHANES questionnaire/medical-condition information. PHQ−9 was calculated from the nine depression items. eGFR was calculated based on serum creatinine, age, and gender using a race-free creatinine-based equation. Physical activity was represented using the available NHANES activity variables. Prescription medication information was used to identify anticonvulsant exposure.

All models retained the NHANES multistage probability sampling structure, including examination weights, strata, and primary sampling units. Odds ratios (ORs), 95% confidence intervals (CIs), and two-sided *p* values were reported. Restricted cubic spline (RCS) analyses were repeated using the same expanded adjustment sets to evaluate whether the nonlinear exposure-response relationship was materially altered.

### 2.3. In Silico Hepatic GGT1 Perturbation and Single-Cell Transcriptomics

Serum GGT activity is associated with the hepatic *GGT1* gene family, with hepatic *GGT1* representing a major hepatic isoform [30]. To explore candidate hepatic transcriptional changes that may be related to hepatic *GGT1* perturbation, we applied scTenifoldKnk [31] to a publicly available human liver single-cell RNA-seq dataset (GEO accession GSE124395; *n* = 12,054 cells). After quality control (nFeature_RNA > 200, percent.mt < 20), data were log-normalized and scaled. Cholangiocytes (clusters 5–8) were selected because of their relatively high endogenous hepatic *GGT1* expression. Differentially regulated genes (DRGs) following virtual hepatic *GGT1* knockout were identified at FDR < 0.05. This computational perturbation was treated as a hypothesis-generating analysis and was not assumed to reproduce changes in serum GGT activity or to establish a causal liver–brain pathway.

### 2.4. Downstream Functional and Network Analyses

Gene Ontology (GO) enrichment of DRGs was performed using clusterProfiler (v4.14.6) [32]. Co-expression analysis and Spearman correlations between hepatic *GGT1* and the identified DRG module were evaluated in GTEx liver data (v11, *n* = 226). Transcription factor enrichment was conducted using TRRUST v2 [33]. Mediation analysis was used to assess whether the observed statistical relationships were compatible with an indirect association involving the candidate transcription factor identified through enrichment analysis; this analysis was interpreted as exploratory and not as proof of molecular mediation. Weighted Gene Co-expression Network Analysis (WGCNA) was performed on GTEx liver data to identify co-expression modules associated with the hepatic *GGT1*-related gene signature and to characterize pathway enrichment, including oxidative stress-related processes.

## 3. Results

### 3.1. 5xFAD Mice Exhibit Progressive Cognitive and Hepatic Phenotypes

**Figure 1:**
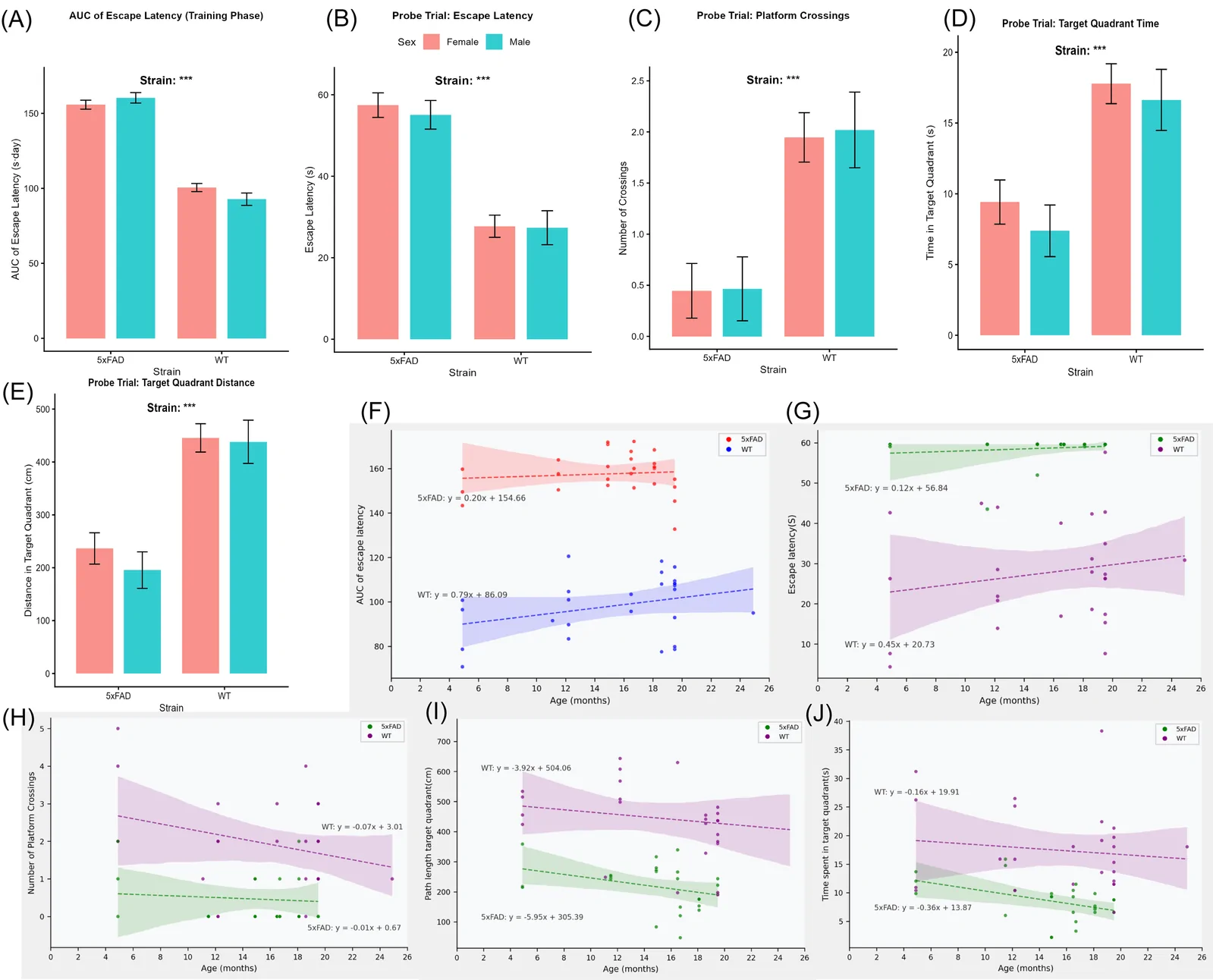
Progressive spatial learning and cognition deficits in *5xFAD* mice. (**A**) Escape latency during the 4-day learning phase of the MWM. The area under the curve (AUC) is shown in the illustration. (**B**) Latency to reach the previous platform location during the probe test. (**C**) Number of platform crossings. (**D**) Time spent in the target quadrant. (**E**) Distance swum in the target quadrant. Data are presented as mean ± SEM. *** *p* < 0.001 compared with wild-type (WT). (**F**–**J**) Age-stratified linear regression analysis showing the relationship between age and MWM performance: (**F**) AUC of escape latency during the 4-day learning phase, (**G**) escape latency, (**H**) number of platform crossings, (**I**) time in target quadrant, (**J**) distance in target quadrant. Shaded areas indicate 95% confidence intervals. *5xFAD* mice exhibit faster age-related cognitive decline compared to *WT* littermates. *n* = 26 per group.

We first characterized the cognitive phenotype. In the Morris water maze (MWM), compared with *WT*littermates, *5xFAD* mice showed impaired spatial learning, reflected by a higher area under the curve (AUC) of escape latency during the 4-day acquisition phase (*p* < 0.001; Figure 1A). During the probe trial, *5xFAD* mice also showed longer latency to the former platform location (*p* < 0.001; Figure 1B), together with differences in other probe-trial measures (Figure 1C–E). No significant sex effects or strain × sex interactions were observed for the reported measures (all *p* > 0.05, Table S6).

Age-stratified linear regression showed different age-related trajectories between strains. Compared with *WT* mice, 5xFAD mice showed a steeper age-related increase in escape latency and greater declines in target-quadrant measures (Figure 1F–J). These analyses describe progressive, strain-associated cognitive differences but do not by themselves establish a causal relationship between hepatic markers and cognition. Because swim speed and visible-platform performance were not assessed, these MWM findings should be regarded as exploratory and cannot be attributed exclusively to spatial cognitive differences.

Parallel histopathological analysis of liver tissue from 4.9 to 19.5 months showed that hepatic abnormalities appeared earlier and were more pronounced in *5xFAD* mice. Although WT mice developed some hepatocyte edema and mild inflammation at advanced ages, *5xFAD* mice showed earlier evidence of grade 4 edema, grade 1 necrosis, and inflammatory infiltration at 16.5 months (Figures S3 and S4; Table S7). The temporal co-occurrence of hepatic and cognitive phenotypes is compatible with an altered liver–brain relationship in this model, but does not by itself demonstrate a liver-to-brain causal pathway.

### 3.2. Strain-Related Differences in Liver Markers and Their Associations with Cognition

**Figure 2:**
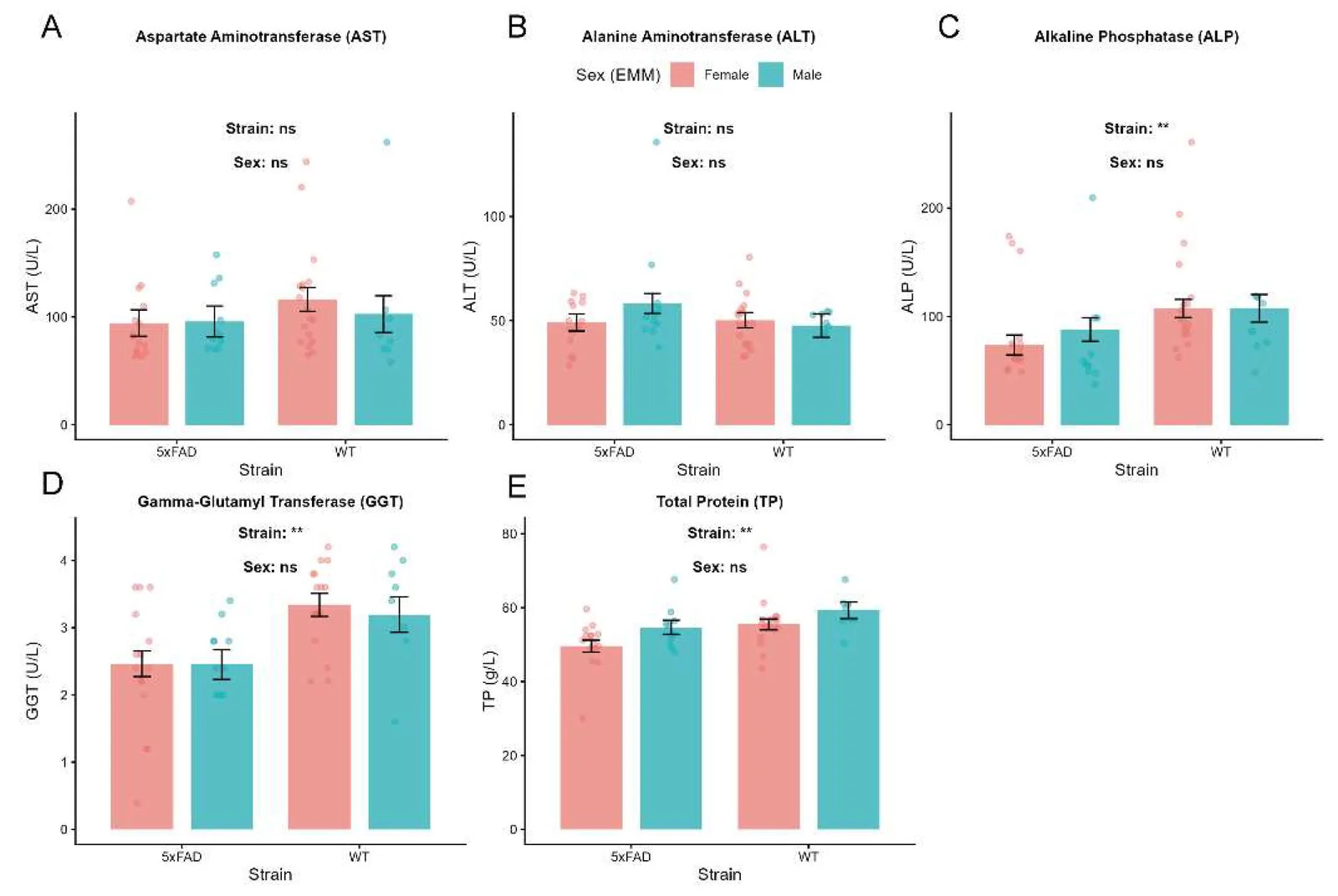
Effects of sex and strain on serum liver function markers in mice after adjusting for age. (**A**–**E**) Bar graphs show estimated marginal means (EMMs) ± SEM of five serum liver biochemical parameters derived from linear regression models adjusting for age (months): (**A**) aspartate aminotransferase (AST), (**B**) alanine aminotransferase (ALT), (**C**) alkaline phosphatase (ALP), (**D**) gamma-glutamyl transferase (GGT), and (**E**) total protein (TP). Individual points represent raw data from single mice. Statistical analysis was performed using two-way ANOVA (strain × sex) with age as a covariate. ** *p* < 0.01 for main effect of strain. *n* = 52 mice (26 WT, 26 5xFAD). ns: no *.

After adjustment for age, multi-factor ANOVA showed significant strain effects for ALP, GGT, and TP, with lower levels in *5xFAD* mice than in *WT* controls (Figure 2C–E). AST and ALT did not differ significantly by strain, and no significant age × strain interactions were observed (Table S8). These findings indicate that several serum liver markers differ between strains in parallel with the cognitive phenotype.

We next examined associations between serum liver markers and cognitive measures. Hierarchical partial correlation analysis (Table 1) showed that higher ALP, GGT, and TP were associated with better MWM performance in unadjusted and partially adjusted models. For GGT, higher serum levels were associated with shorter escape latency (r = −0.332, *p* = 0.02) and greater target-quadrant distance (r = 0.344, *p* = 0.01) in the probe trial. After adjustment for strain, most GGT–cognition associations were attenuated and became non-significant. However, a positive association between GGT and platform crossings emerged in Model 3 (r = 0.321, *p* = 0.02). These changes indicate that between-strain differences strongly influence the observed correlations, and that strain adjustment may introduce collinearity or overadjustment. Thus, the observed mouse-level correlations appear to be substantially influenced by between-strain differences, and the mouse data should be interpreted as associative rather than evidence that GGT independently predicts cognition within genotype.

### 3.3. Higher Serum GGT Is Inversely Associated with the LCP in Humans

Among 3181 NHANES participants, 417 met the study-defined LCP criterion and 2764 were classified as normal cognition (NC). Compared with the NC group, participants in the LCP group were older, had lower educational attainment, were more likely to be widowed or divorced, and were more often non-drinkers. Median GGT levels were lower in the LCP group than in the NC group, and median ALP levels were also lower in the LCP group (Table S9).

Next, we evaluated associations between liver markers and LCP using three nested logistic regression models (Table 2). In the unadjusted model, both GGT and ALP showed inverse associations with the phenotype. Compared with Q1, the highest GGT quartile (Q4) had an OR of 0.53 (*p* < 0.001). After adjustment for demographic factors in Model 2, the association was attenuated but remained statistically significant (Q4 OR = 0.61, *p* < 0.001). In the fully adjusted Model 3 (without LDL), the inverse association remained (Q4 OR = 0.54, *p* < 0.001). In the fasting-subsample sensitivity analysis using WTSAF2YR and additional adjustment for LDL, the inverse association remained statistically significant (Q4 vs. Q1 OR = 0.63, *p* < 0.001). The attenuation of the point estimate and the wider confidence interval are consistent with the smaller sample size and the additional adjustment for LDL, and do not alter the overall direction or significance of the association (Table S10). These estimates indicate lower odds, rather than lower absolute risk, of the study-defined phenotype among participants in the highest GGT quartile relative to Q1.

In the primary multivariable model (Model 3), participants in the highest GGT quartile had significantly lower odds of low cognitive performance than those in the lowest quartile (OR, 0.54; *p* < 0.001). As shown in Table S11, in the expanded-adjustment analyses, the association remained directionally consistent and statistically significant after sequential adjustment for additional potential confounders. The corresponding ORs for Q4 versus Q1 were 0.55 (*p* < 0.001) in Model 4, 0.58 (*p* < 0.001) in Model 5, and 0.59 (*p* < 0.001) in Model 6. We also assessed multicollinearity among the model predictors and fitted an expanded sensitivity model including the other liver markers. The VIF/GVIF results are presented in Table S12.

In contrast, the inverse association observed for ALP in the unadjusted model was attenuated and became non-significant after covariate adjustment. As shown in Table S13, In Model 1, Q4 ALP was associated with lower odds of the phenotype (OR = 0.59, *p* < 0.001), whereas the association was not significant in Model 2 (OR = 0.84, *p* = 0.123) or Model 3 (OR = 0.93, *p* = 0.533). No significant associations with the study-defined phenotype were observed for AST, ALT, or TP across the models (Tables S14–S16). Within the set of markers examined, GGT showed the most persistent inverse association in the adjusted human analyses.

### 3.4. Nonlinear Association Between Serum GGT and LCP

**Figure 3:**
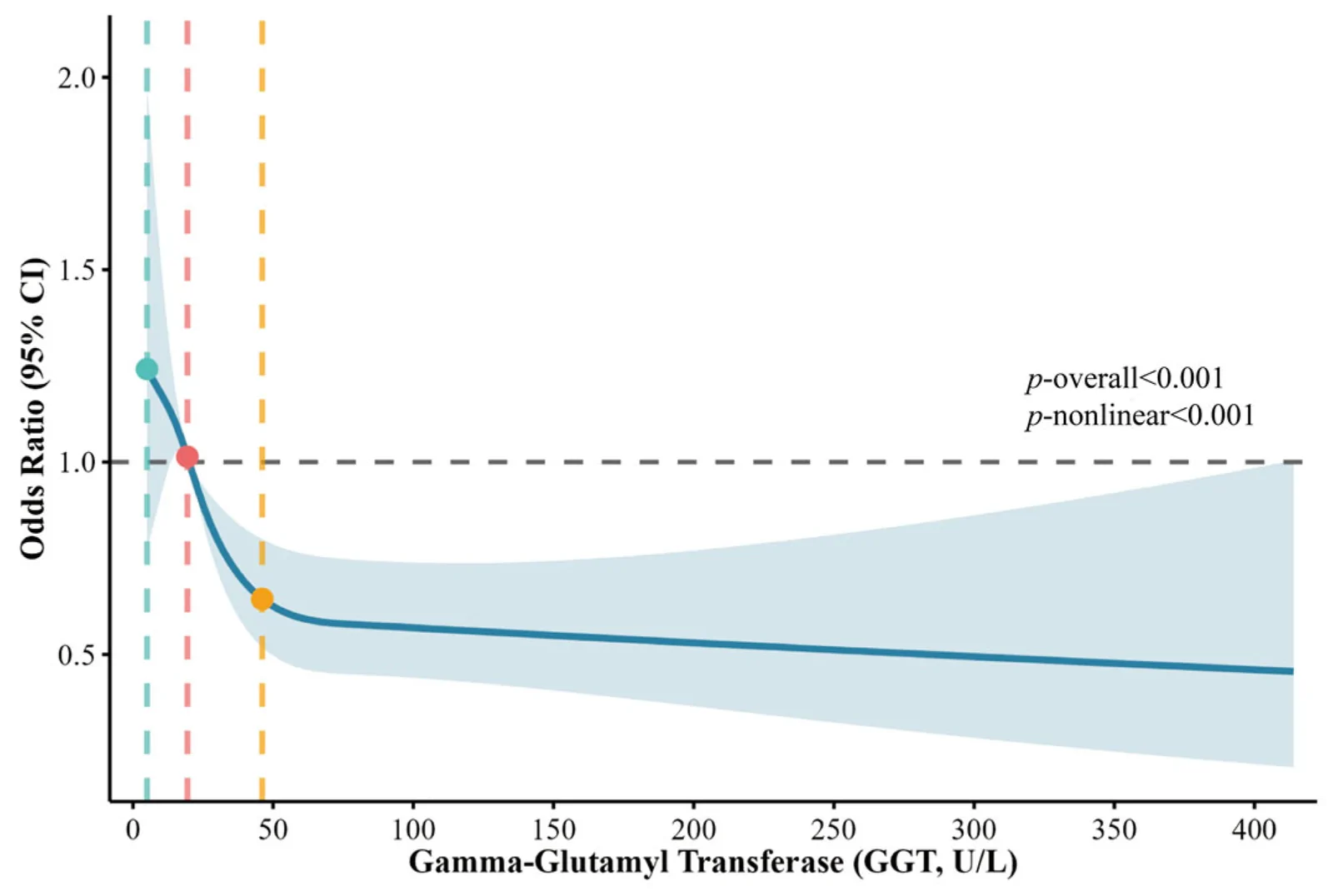
Nonlinear association between serum GGT levels and LCP risk in humans. RCS analysis with three knots was used to model the nonlinear association between serum GGT and LCP in NHANES participants (*n* = 3181). The primary model was adjusted for age, gender, education level, marital status, BMI, alcohol consumption, HDL, and sleep duration. Other liver markers (AST, ALT, ALP, TP) and LDL were not included in the primary model because of potential overadjustment collinearity. The reference value was set at the model-derived inflection point of 19.4 U/L (red dot). The inflection point is presented as a feature of the fitted association and requires external validation prior to clinical interpretation.

RCS analysis identified a statistically significant nonlinear association between serum GGT and the odds of LCP (*p*-nonlinear < 0.001; Figure 3). With the reference set at 19.4 U/L, the estimated OR was 1.0 at that value; at 5.0 U/L, the estimated OR was 1.32, while at approximately 46 U/L, the estimated OR was about 0.70. The 19.4 U/L value is a data-derived inflection point within this model and should not be interpreted as a clinical diagnostic or treatment threshold. When adjusting to other models, its turning point does not change much (Table S17). The low-GGT estimate also had a confidence interval that included the null, indicating uncertainty in that portion of the curve.

### 3.5. Subgroup Analyses of the GGT–Cognition Association

To examine whether the association varied across population subgroups, GGT was categorized using clinical cut-points: low (<10 U/L), normal (10–60 U/L, reference), and high (≥60 U/L). High GGT was associated with lower odds of LCP in the overall population (OR = 0.67, *p* = 0.001; Table S18). Similar inverse associations were observed in participants aged ≤65 years (OR = 0.62, *p* = 0.001), males (OR = 0.57, *p* = 0.002), normal BMI (18.5–24.9 kg/m^2^; OR = 0.48, *p* = 0.009) and long sleep duration (OR = 0.60, *p* = 0.003). However, subgroup-specific estimates should be interpreted cautiously because subgroup analyses may have lower precision and are observational. Age, gender, education, marital status, alcohol consumption, BMI, and sleep duration showed no statistically significant interactions.

### 3.6. In Silico Hepatic GGT1 Perturbation Identifies an Immunoglobulin-Related Transcriptional Signature

**Figure 4:**
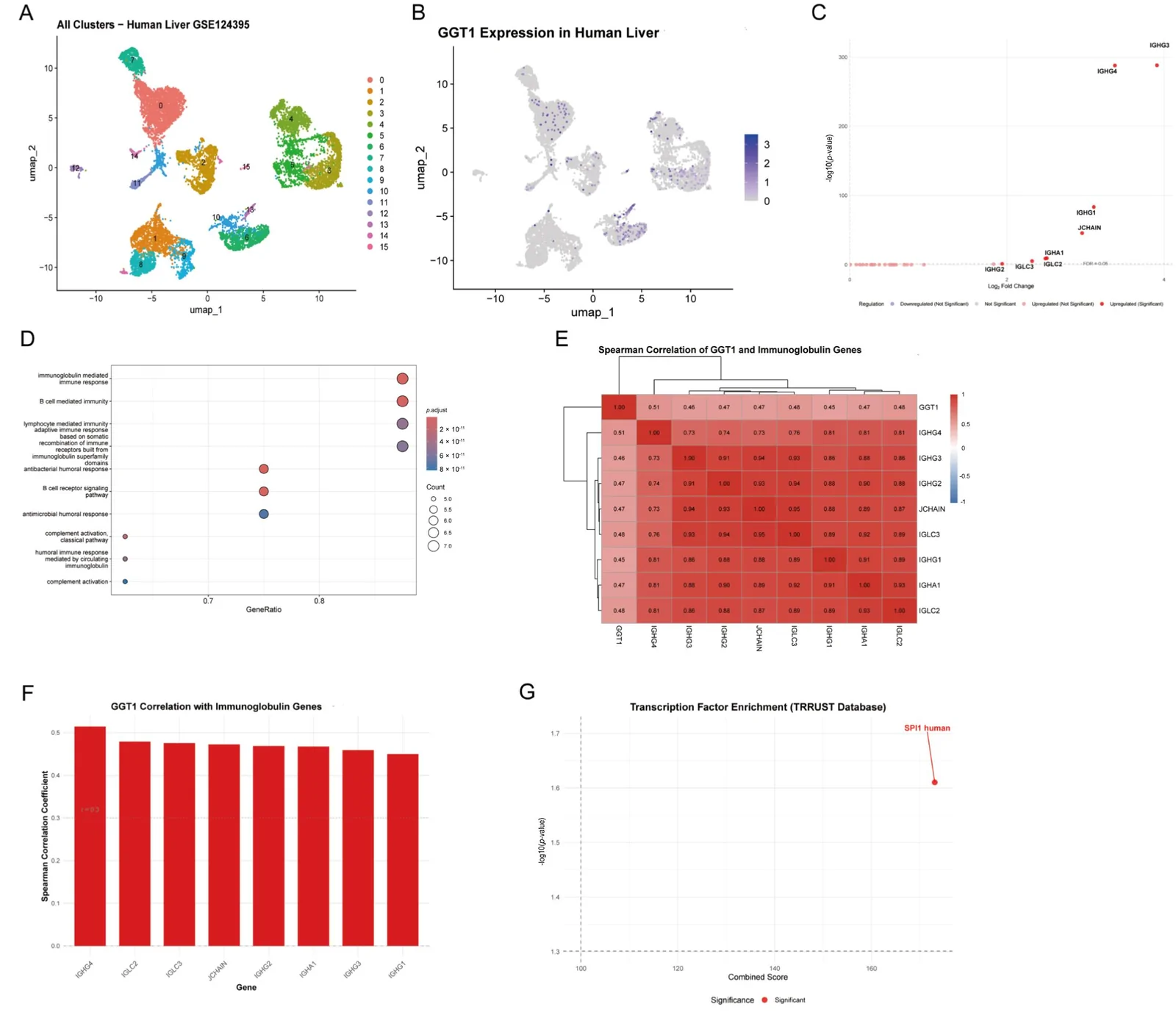
Hepatic *GGT1* virtual knockout identifies an immunoglobulin-related hepatic transcriptional signature. (**A**) Clustering map of human liver single-cell data. (**B**) Hepatic *GGT1* expression in the cholangiocyte population. (**C**) Virtual hepatic *GGT1* knockout identified 8 significantly upregulated genes (FDR < 0.05), all belonging to the immunoglobulin-related gene family (IGHG1-4, IGHA1, IGLC2, IGLC3, JCHAIN), with no significantly downregulated genes. (**D**) GO enrichment analysis showed enrichment in immunoglobulin-mediated immune response and B cell-mediated immune pathways (fold enrichment = 78.4, *p* < 0.001). (**E**) GTEx liver data showed strong co-expression among the 8 Ig-related genes (average pairwise r = 0.86). (**F**) Hepatic *GGT1* was positively correlated with the Ig module (r = 0.45–0.51, all *p* < 0.001). (**G**) TRRUST transcription factor enrichment identified *SPI1* (PU.1) as a significantly enriched upstream regulator (combined score = 173.1, *p* = 0.025). These analyses are computational and should be interpreted as hypothesis-generating rather than as direct evidence of regulatory function.

We emphasize that serum GGT activity is not equivalent to hepatic *GGT1* expression or activity. The following analyses are computational and exploratory, and should not be interpreted as mechanistic evidence for the association between serum GGT and cognition. As shown in Figure 4B, hepatic *GGT1* was highly expressed in the cholangiocyte population. To explore candidate transcriptional changes associated with hepatic *GGT1* perturbation, we performed in silico hepatic *GGT1* knockout in the human liver single-cell atlas. Eight genes were significantly predicted to be upregulated following virtual knockout (FDR < 0.05), all of which were immunoglobulin-related (IGHG1-4, IGHA1, IGLC2, IGLC3, JCHAIN; Figure 4C), and no significantly downregulated genes were identified. GO analysis showed enrichment of B cell-mediated immunity and immunoglobulin-mediated immune response pathways (fold enrichment = 78.4, *p* <0.001; Figure 4D). In GTEx liver data, these genes showed strong co-expression (mean pairwise Spearman r = 0.86, all *p* < 0.001; Figure 4E), and hepatic *GGT1* expression was positively correlated with the Ig module (r = 0.45–0.51, all *p* < 0.001; Figure 4F). Transcription factor enrichment identified *SPI1* (PU.1) as a significantly enriched regulator (composite score = 173.1, *p* = 0.025; Figure 4G). These results identify a coherent computational signature, but they do not establish that hepatic *GGT1* directly regulates immunoglobulin transcription in cholangiocytes or that the signature mediates the association between serum GGT and cognition.

**Figure 5:**
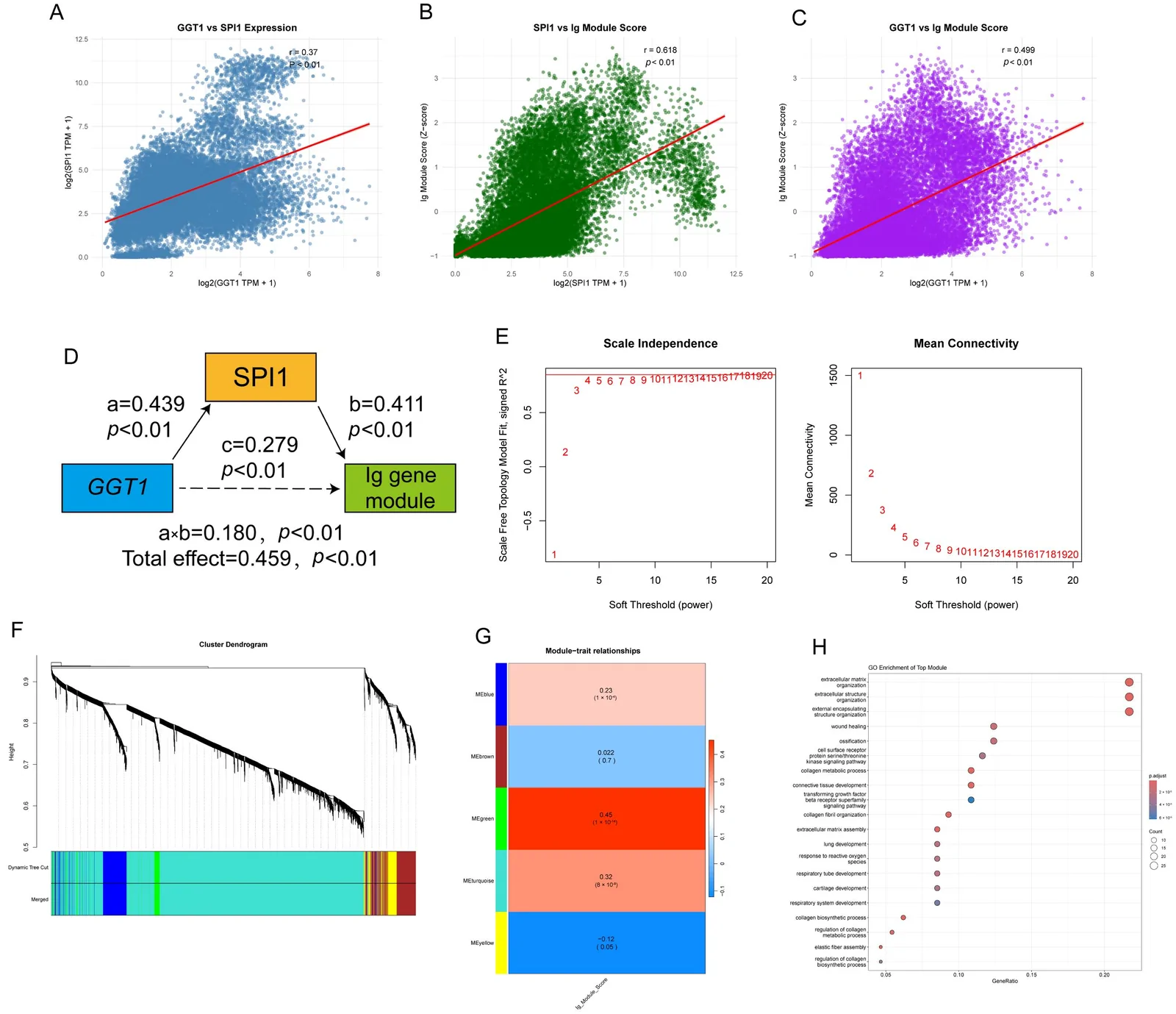
Hypothesis-generating computational relationships among hepatic *GGT1*, *SPI1*, the immunoglobulin-related signature, and hepatic co-expression modules. (**A**) Correlation between hepatic *GGT1* and *SPI1* expression. (**B**) Correlation between *SPI1* and the immunoglobulin-related module. (**C**) Correlation between hepatic *GGT1* and the immunoglobulin-related module. (**D**) Mediation schematic showing the total, direct, and indirect statistical associations among hepatic *GGT1*, *SPI1*, and the immunoglobulin-related module. The indirect effect was 0.180 (*p* < 0.01), corresponding to 39.25% of the total statistical effect. (**E**) WGCNA scale-free topology fit curve (left) and mean connectivity (right). (**F**) WGCNA gene dendrogram with module colors. (**G**) Module–trait association heatmap showing the strongest positive correlation between MEgreen and Ig-related features (r = 0.453, *p* < 0.01). (**H**) GO enrichment of MEgreen genes, including reactive oxygen species metabolic process (*p* < 0.01), oxidative stress response (*p* < 0.01), and extracellular matrix organization. These analyses are computational and associative. They do not establish a directional or causal pathway and are not integrated with the human or mouse association estimates.

Mediation analysis in GTEx liver data showed a statistically significant indirect association involving *SPI1* (indirect effect = 0.180, corresponding to 39.25% of the estimated total statistical effect under the specified model, *p* < 0.001; Figure 5A–D). Because the analysis was based on observational expression data and did not experimentally manipulate hepatic *GGT1* or *SPI1*, the estimated proportion should be interpreted as a model-based statistical decomposition rather than evidence that *SPI1* functionally mediates a hepatic *GGT1*-to-Ig pathway.

WGCNA was then used to characterize the broader transcriptional context of the Ig-related signature. Among five co-expression modules, the green module (MEgreen) showed the strongest positive correlation with the Ig gene signature (r = 0.453, *p* < 0.01 Figure 5G). GO enrichment of MEgreen genes included extracellular matrix organization and oxidative stress-related processes, including reactive oxygen species metabolic process (*p* < 0.01) and response to oxidative stress (*p* < 0.01; Figure 5H). *SOD3* and *CYGB* were among the genes in this module. These results indicate that the Ig-related signature co-occurs with oxidative stress-related transcriptional programs in GTEx liver data, but co-expression and pathway enrichment do not establish a direct regulatory or causal relationship among hepatic *GGT1*, SPI1, immunoglobulin genes, and oxidative stress.

## 4. Discussion

This study examined the relationship between serum GGT and cognitive outcomes across complementary mouse, population, and transcriptomic analyses. In *5xFAD* mice, serum GGT was associated with better cognition in unadjusted and partially adjusted analyses, but these associations were attenuated and became non-significant after adjustment for strain. In the NHANES cohort, serum GGT was inversely associated with the odds of LCP after multivariable adjustment, with an OR of 0.54 for Q4 versus Q1. The association remained after further adjustment, supporting the idea that the negative link between GGT and low cognitive performance is not readily attributable to the measured demographic, socioeconomic, cardiometabolic, behavioral, depressive, renal, or anticonvulsant-related factors. Nevertheless, these analyses would not eliminate the possibility of residual confounding. In particular, some covariates may be imperfectly measured, medication exposure may be incompletely captured, and several factors could lie in the causal pathway rather than functioning exclusively as confounders. Furthermore, the cross-sectional design of NHANES precludes establishing temporality or causality.

Taken together, the findings support an epidemiological association between serum GGT and cognitive performance while also highlighting important differences between species and analytical models [15,34,35]. Although Zhang and others also studied liver enzymes and cognition in NHANES 2011–2014, our study differs in three key ways [34]. First, our analysis sample is larger (*n* = 3181 versus *n* = 2764), giving us more statistical power, especially for nonlinear analyses. Second, our main cognitive outcome is a composite score with a predefined threshold for assessing LCP, while Zhang and colleagues used a single cognitive test score. Third, and most importantly, we integrated mouse behavioral data, human epidemiological data, and liver single-cell transcriptome analysis into a cross-species framework, which they did not attempt.

Several large prospective cohort studies have reported that higher GGT is associated with increased dementia risk [16,36]. A recent meta-analysis similarly found that higher GGT was associated with increased cognitive impairment risk [15]. In contrast, our cross-sectional analysis found an inverse association (Q4 vs. Q1 OR = 0.54) and a lower odds of LCP even at GGT ≥60 U/L (OR = 0.67). Three non-exclusive explanations may reconcile this discrepancy.

First, low GGT may serve as a marker of frailty, malnutrition, or reduced hepatic synthetic reserve rather than reflecting a protective biological effect. GGT is an enzyme involved in glutathione metabolism and its synthesis requires adequate nutritional and metabolic status [12]. In older adults, low GGT levels have been observed in individuals with lower muscle mass and incident cognitive frailty [37,38]. If the highest GGT quartile in our sample contains a relatively healthier subgroup with preserved hepatic synthetic function, the inverse association could be driven by the sicker low-GGT group rather than by a protective effect of GGT itself.

Second, residual confounding by alcohol remains a major concern. The LCP group had a higher prevalence of non-drinkers (52.0% vs. 43.6%), which is consistent with the “sick-quitter” effect, where individuals stop drinking due to declining health. Because alcohol intake is the strongest lifestyle determinant of GGT [39], a higher proportion of sick-quitters in the non-drinking group could simultaneously elevate GGT in the NC group and concentrate frail individuals in the LCP group, creating a spurious inverse association. Conversely, the “healthy-drinker” effect could make moderate drinkers appear healthier than they are, further complicating the interpretation. Although we adjusted for alcohol consumption, single-measurement assessment cannot distinguish lifetime abstainers from sick-quitters.

Third, cross-sectional design limits causal direction. The prospective studies cited above measured GGT years before dementia diagnosis [16], whereas our study assessed GGT and cognition at a single timepoint. Reverse causation—whereby preclinical cognitive decline leads to frailty, malnutrition, and reduced GGT—remains possible.

The other liver markers showed less consistent patterns. AST and ALT were not significantly associated with the cognitive phenotype across the principal analyses. TP showed positive correlations with several mouse cognitive measures but no corresponding association in the human cohort. ALP showed an inverse association in the unadjusted human analysis that was substantially attenuated after covariate adjustment. These differences suggest that liver-related biomarkers may capture distinct physiological processes and that crude associations can be sensitive to demographic, metabolic, and hepatic covariates. Importantly, the present data do not establish that GGT is uniquely protective; rather, among the markers examined, GGT showed the most persistent inverse association in the adjusted human analyses.

Several biological mechanisms could plausibly connect GGT-related physiology with cognition, including glutathione metabolism, oxidative stress, inflammation, and lipid-related pathways [6,40,41]. However, serum GGT should be regarded as a biomarker of systemic and hepatic physiology rather than a direct surrogate for hepatic *GGT1* activity. Elevated GGT can occur in multiple metabolic, hepatic, inflammatory, and lifestyle contexts, and its biological interpretation is therefore complex [40,41,42]. The present study was not designed to distinguish whether the observed association reflects a direct biological effect of GGT, a compensatory response, or residual confounding. These alternative explanations should be considered when interpreting the liver–brain axis hypothesis [6,9,43].

The single-cell analyses provide a candidate molecular signature that may warrant further investigation. Virtual hepatic *GGT1* knockout in cholangiocytes identified eight immunoglobulin-related genes, which were strongly co-expressed in GTEx liver data and showed associations with SPI1. Exploratory mediation analysis was statistically compatible with an indirect relationship involving SPI1, while WGCNA placed the Ig-related signature within a broader module enriched for oxidative stress-related pathways. Nevertheless, these analyses rely on computational perturbation and observational transcriptomic data [44,45]. In particular, the concentration of immunoglobulin transcripts among the virtual knockout hits warrants careful assessment of cell-type composition, ambient RNA, doublets, and annotation quality before a direct cholangiocyte regulatory mechanism is inferred [46,47,48]. Thus, the hepatic *GGT1*–*SPI1*–Ig–oxidative stress relationship should be considered a testable hypothesis rather than an established mechanistic pathway.

The nonlinear association observed in NHANES also requires cautious interpretation. The model-derived inflection point of approximately 19.4 U/L is a feature of the fitted cross-sectional association and should not be interpreted as a biologically established threshold or a clinical treatment target [34]. Likewise, the apparent plateau around 46 U/L describes the shape of the fitted curve rather than a validated range of cognitive benefit. Differences in population characteristics, assay methods, covariate distributions, and cognitive definitions could shift these values in other cohorts [15,34]. Prospective studies with repeated GGT measurements and clinically meaningful cognitive outcomes are needed to determine whether the nonlinear pattern is reproducible.

Subgroup analyses suggested inverse associations in participants aged ≤65 years, males, and those with normal BMI. However, these subgroup-specific estimates should be regarded as exploratory. The age interaction *p* value of 0.065 does not meet the conventional threshold for statistical significance, so the current data do not establish effect modification by age. Differences in subgroup estimates may reflect heterogeneity in sample size, residual confounding, or chance. Future studies should prespecify effect-modification hypotheses and test them in adequately powered cohorts [49].

The three components of this study should be interpreted as parallel observations rather than as an integrated mechanistic pathway. The virtual hepatic *GGT1* knockout predicts that loss of hepatic *GGT1* function leads to upregulation of immunoglobulin-related genes in cholangiocytes. If hepatic *GGT1* activity is positively correlated with serum GGT, this would predict that lower serum GGT is associated with higher hepatic immunoglobulin-related transcriptional programs. In the human analysis, lower serum GGT was associated with higher odds of LCP, which is directionally consistent with the hypothesis that low hepatic *GGT1* activity and upregulated immunoglobulin-related programs accompany poorer cognitive performance. However, this apparent consistency does not demonstrate mediation, because serum GGT is not equivalent to hepatic *GGT1* activity, and the virtual knockout is computational rather than experimental.

No current dataset in this study can directly test the proposed directional hypothesis, because hepatic transcriptomic data, serum GGT, and cognitive measurements were not available in the same individuals or animals. The mouse data do not provide a clear test of this hypothesis. *5xFAD* mice had lower serum GGT than *WT* mice and poorer cognition, but after adjustment for strain, GGT was not consistently associated with cognitive outcomes within genotype. Thus, the mouse data are compatible with a strain-level association but do not establish a within-genotype GGT–cognition link. Future studies could test this hypothesis by performing liver-specific *GGT1* knockout or overexpression in mice, followed by measurement of hepatic immunoglobulin-related transcripts, serum GGT, and cognitive outcomes.

### 4.1. Interpretation and Potential Translational Relevance

GGT is an inexpensive and routinely available serum biomarker, which makes the observed association potentially relevant to future risk-stratification research. However, the current data are insufficient to support use of GGT as a stand-alone cognitive risk marker. The approximately 19 U/L inflection point is model-derived and has not been externally validated; it should not be presented as a clinically actionable threshold. Similarly, the observed inverse association does not establish that increasing GGT would reduce cognitive risk. Before clinical application can be considered, the association should be replicated in independent prospective cohorts, evaluated against clinically adjudicated cognitive outcomes, and assessed for incremental predictive value beyond established risk factors and biomarkers [50,51].

The transcriptomic findings provide hypotheses for future mechanistic work rather than a basis for therapeutic intervention. Experimental studies using targeted manipulation of hepatic *GGT1* and *SPI1* in relevant hepatic cell models, together with measurement of immunoglobulin expression, redox status, circulating mediators, and cognitive outcomes in vivo, would be needed to determine whether the proposed relationships are functional. Similarly, studies should distinguish the biological consequences of changing hepatic *GGT1* activity from simply changing GGT concentrations. Until such evidence is available, lifestyle or pharmacological strategies aimed at increasing GGT should not be inferred from the present results [52,53].

### 4.2. Limitations

Several limitations should be considered. First, the NHANES analysis is cross-sectional, so temporality and causality cannot be established. Reverse causation and residual confounding remain possible despite multivariable adjustment [54]. Second, the mouse correlations were substantially attenuated after adjustment for strain, indicating that between-strain differences are an important source of the observed covariance. Third, the virtual knockout, transcription factor enrichment, mediation, and WGCNA analyses are computational or observational and require experimental validation. Fourth, LDL-cholesterol was available only in the morning fasting subsample. To avoid weight inconsistency, LDL was not included in the primary full-sample model; its influence was evaluated in a fasting-subsample sensitivity analysis using WTSAF2YR. The sensitivity analysis had a smaller sample size and therefore had lower precision than the primary analysis.

## 5. Conclusions

Serum GGT was inversely associated with LCP in NHANES, whereas the corresponding association in *5xFAD* mice was attenuated after adjustment for strain. The transcriptomic analyses identified an immunoglobulin-related hepatic signature associated with *SPI1* and oxidative stress-related pathways. These findings are parallel and hypothesis-generating; they do not establish a mechanistic liver–brain pathway. A testable directional hypothesis is that lower hepatic *GGT1* activity or lower serum GGT is associated with upregulated cholangiocyte immunoglobulin-related programs and poorer cognitive performance. Testing this hypothesis will require experimental manipulation of hepatic *GGT1* and *SPI1* in hepatic cells and in vivo, together with paired hepatic transcriptomic, serum GGT, and cognitive measurements.

## Figures and Tables

**Table 1:** Stepwise partial correlation analysis between serum liver function markers and cognitive performance in mice.

Indicator	MWM Outcome	Model 1	Model 2	Model 3
		Partial r	Partial r	Partial r
AST	EL	−0.255	−0.251	−0.18
	PC	0.322 *	0.324 *	0.275
	TQT	0.195	0.184	0.095
	TQD	0.220	0.211	0.119
ALT	EL	0.066	0.071	−0.036
	PC	−0.013	−0.014	0.137
	TQT	−0.136	−0.127	−0.088
	TQD	−0.092	−0.090	−0.059
ALP	EL	−0.291 *	−0.295 *	−0.033
	PC	0.398 **	0.392 **	0.193
	TQT	0.114	0.117	−0.073
	TQD	0.126	0.122	−0.005
GGT	EL	−0.332 *	−0.334 *	0.143
	PC	0.063	0.097	0.321 *
	TQT	0.206	0.186	−0.182
	TQD	0.344 *	0.313 *	−0.088
TP	EL	−0.387 **	−0.382 **	−0.166
	PC	0.338 *	0.252	0.166
	TQT	0.198	0.192	0.008
	TQD	0.350 *	0.355 *	0.186

Abbreviations: EL—escape latency (probe trial); PC—platform crossings; TQT—target quadrant time; TQD—target quadrant distance; Model 1—unadjusted Pearson correlation; Model 2—partial correlation controlling for age (months) and sex; Model 3—partial correlation controlling for age, sex, and strain. * *p* < 0.05, ** *p* < 0.01.

**Table 2:** Association between GGT quartiles and LCP in the NHANES cohort.

Group	Characteristic	Range	*n*	OR	95% CI	*p*
Model 1	GGT4					
	Q1	<14	902	—	—	
	Q2	14–20	835	0.74	0.59, 0.93	0.011
	Q3	21–30	764	0.62	0.49, 0.78	<0.001
	Q4	≥31	680	0.53	0.43, 0.66	<0.001
Model 2	GGT4					
	Q1	<14	902	—	—	
	Q2	14–20	835	0.86	0.68, 1.09	0.204
	Q3	21–30	764	0.71	0.56, 0.90	0.005
	Q4	≥31	680	0.61	0.49, 0.77	<0.001
Model 3	GGT4					
	Q1	<14	902	—	—	
	Q2	14–20	835	0.85	0.67, 1.08	0.184
	Q3	21–30	764	0.69	0.54, 0.88	0.003
	Q4	≥31	680	0.54	0.42, 0.70	<0.001

Note: Abbreviations: GGT4—γ-glutamyl transferase quartiles; OR—odds ratio; CI—confidence interval. Unweighted *n* refers to the number of participants in each quartile. Model 1: Unadjusted. Model 2: Adjusted for demographic factors (age, gender, education, marital). Model 3 (primary): Further adjusted for BMI, alcohol consumption, HDL, and sleep duration. Other liver markers (AST, ALT, ALP, TP) and LDL were not included in the primary model. The primary Model 3 did not include LDL-cholesterol because LDL is available only in the fasting subsample. A sensitivity analysis including LDL, using fasting subsample weights (WTSAF2YR), is presented in Table S10. The first quartile (Q1) was used as the reference group. Statistical significance was defined as *p* < 0.05.

## Data Availability

Detailed human data can be accessed publicly at https://www.cdc.gov/nchs/nhanes/ (accessed on 5 December 2025); the single-cell transcriptome data comes from https://www.ncbi.nlm.nih.gov/geo/ (accessed on 13 January 2026), and the GTEx data can be downloaded from https://www.gtexportal.org/home/ (accessed on 20 January 2026). Other data are available from the authors upon reasonable request.

## Supplementary Materials

The following supporting information can be downloaded at: https://www.mdpi.com/article/10.3390/life16101735/s1, Figure S1: Flowchart of participant selection from the NHANES cohort; Figure S2: Directed acyclic graph (DAG) of the relationship between serum GGT and LCP; Figure S3: Age-related hepatic histopathology in 5xFAD and WT mice; Figure S4: Remaining H&E-stained liver sections from 5xFAD and WT mice of different ages.; Table S1: Target platform and change in mouse starting position; Table S2 Semi-quantitative grading scale for histopathological assessment of mouse liver tissue; Table S3: Characteristics of included and excluded participants by reason for exclusion; Table S4: Quartile cut-points for serum liver markers in the NHANES 2011–2014 analytic sample; Table S5: Definition of covariates across models; Table S6: Effects of age and gender on learning and probe trial performance in the MWM; Table S7: Individual histopathological scoring of liver tissue from wild-type (WT) and 5xFAD mice across different ages; Table S8 Statistical analysis of liver function-related serum biochemical parameters; Table S9 Demographic and clinical characteristics of NHANES participants by memory status; Table S10: Sensitivity analysis of the association between serum GGT quartiles and LCP in the morning fasting subsample; Table S11 Expanded-adjustment sensitivity analyses; Table S12: Collinearity diagnostics for the multivariable model; Table S13: Multivariate logistic regression analysis of the association between ALP and LCP in the population; Table S14: Multivariate logistic regression analysis of the association between ALT and LCP in the population; Table S15: Multivariate logistic regression analysis of the association between AST and LCP in the population; Table S16: Multivariate logistic regression analysis of the association between TP and LCP in the population; Table S17: Nonlinear association between serum GGT and LCP across models; Table S18: Subgroup analysis of the association between serumGGT and LCP.

## Abbreviations

AD	Alzheimer’s disease
LCP	Low cognitive performance
NC	Normal cognition
MWM	Morris Water Maze
EMM	Estimated Marginal Means
AST	Aspartate aminotransferase
ALT	Alanine aminotransferase
ALP	Alkaline phosphatase
GGT	Gamma-glutamyl transferase
TP	Total protein
RCS	Restricted cubic splines
OR	Odds ratio
EL	Escape latency
PC	Platform crossings
TQT	Target quadrant time
TQD	Target quadrant distance
GGT4	γ-glutamyl transferase quartiles
