# Supplementary material for: Gamma-Glutamyl Transferase and Cognitive Outcomes: A Cross-Species Analysis and Hepatic Transcriptomic Signature
Source: Life (Basel). 2026 Oct 16;16(10):1735. doi: 10.3390/life16101735 (PMC13706570; doi:10.3390/life16101735)
Supplement: Supplementary file 1 [file life-16-01735-s001.zip › life-4604073-supplementary.pdf]

Table S1 Target platform and change in mouse starting position.

Count		1 day	2 day	3 day	4 day	5 day
	Target platform position	The starting position of the mouse changed as follows				The target platform is removed from the starting position of the mice as follows.
1	III	I	II	IV	III	I
2		II	I	III	IV	
3		IV	III	II	I	
4		III	IV	I	II	

Note: The starting position of the mouse entering the water each day. I: First quadrant; II: Second quadrant; III: Third quadrant; IV: Fourth quadrant.

Table S2 Semi-quantitative grading scale for histopathological assessment of mouse liver tissue.

Grade(number)	Types	explanation
0	Within the normal range	Under study conditions, considering factors such as the animal's age, sex, and strain, the tissue is considered normal. Changes occurring under other conditions can be regarded as abnormal.
1	Very slight	The changes that occurred just exceeded the normal range
2	Slight	Lesions can be observed, but they are not yet severe.
3	Medium	The lesion is obvious and is likely even more severe.
4	serious	The lesion is very severe (the lesion has occupied the entire tissue or organ)

Histopathological changes in mouse liver tissue were graded semi-quantitatively according to the INHAND (International Harmonization of Nomenclature and Diagnostic Criteria) guidelines. A five-grade scale (0–4) was used to assess the severity of hepatocyte edema, inflammatory cell infiltration, hepatocyte necrosis, and connective tissue proliferation (fibrosis).

Table S3 Characteristics of included and excluded participants by reason for exclusion.

Characteristic	Included (n = 3,181)	Excluded due to missing blood data (n = 2,472)	Excluded due to missing cognitive data (n = 1,976)	Excluded due to missing demographic data (n = 609)	p-value
n					
**Age** (median)	68.0 [62.0, 75.0]	69.0 [63.0, 76.0]	70.0 [64.0, 77.0]	69.0 [63.0, 76.0]	<0.001
**Gender** (%)					0.12
Female	1643 (51.7)	1310 (52.9)	1028 (51.9)	317 (52.0)	
Male	1538 (48.3)	1162(47.1)	948(48.1)	292(48.0)	
**Education** (%)					0.08
Never attended school	397 (12.5)	346 (13.9)	296 (13.8)	NA	
Elementary school	689 (21.7)	544 (22.1)	454 (22.9)	NA	
Middle school	858 (27.0)	667 (26.8)	514 (26.3)	NA	
High school	742 (23.3)	544 (22.2)	435 (22.1)	NA	
College or above	495 (15.6)	371 (15.0)	277 (14.9)	NA	
**Marital** (%)					0.15
Divorced	306 (9.6)	247 (10.1)	198 (10.2)	NA	
Married	1789 (56.2)	1335 (53.9)	1047 (52.8)	NA	
Never married	263 (8.3)	198 (8.1)	158 (8.1)	NA	
Partner	182 (5.7)	136 (5.5)	109 (5.6)	NA	
Separated	149 (4.7)	124 (4.9)	99 (5.0)	NA	
Widowed	492 (15.5)	432 (17.5)	366 (18.3)	NA	
**Daily drinks**					0.06

(%)					
Mild	1582 (49.7)	1112 (45.2)	869 (44.1)	NA	
Heavy	177 (5.6)	223 (8.9)	158 (8.0)	NA	
None	1421 (44.7)	1137 (45.9)	948 (47.9)	NA	
**BMI** (%)					0.09
18.5-24.9	937 (29.5)	692 (28.1)	563 (28.3)	177 (29.2)	
<18.5	20 (0.6)	20 (0.7)	14 (0.8)	3 (0.5)	
25.0–29.9	2063 (64.9)	1607 (65.0)	1284 (65.0)	396 (64.7)	
≥30.0	161 (5.1)	153 (6.3)	115 (5.9)	33 (5.6)	
**HDL (median)**	50.0 [40.0, 60.0]	NA			
**AST (median)**	22.0 [18.0, 27.0]	NA	22.0 [18.0, 27.0]	22.0 [18.0, 27.0]	0.30
**ALT (median)**	20.0 [15.0, 28.0]	NA	20.0 [16.0, 28.0]	21.0 [16.0, 29.0]	0.40
**ALP (median)**	69.0 [57.0, 84.0]	NA	70.0 [58.0, 85.0]	69.0 [57.0, 84.0]	0.10
**GGT (median)**	21.0 [14.0, 31.0]	NA	21.0 [14.0, 31.0]	21.0 [13.0, 31.0]	0.05
**TP (median)**	7.1 [6.8, 7.4]	NA	7.2 [6.8, 7.4]	7.1 [6.8, 7.4]	0.20
**Sleep hours (median)**	6.5 [5.5, 7.5]	6.5 [5.5, 7.5]	6.5 [5.5, 7.5]	6.5 [5.5, 7.5]	0.15

Note: Data are weighted median [IQR] for continuous variables and weighted percentage for categorical variables. NA = not available because participants were excluded precisely because the corresponding data were missing. Comparisons used survey-weighted t-tests or Rao–Scott chi-square tests, incorporating 4-year MEC examination weights (WTMEC2YR/2), strata (SDMVSTRA), and PSUs (SDMVPSU).

Table S4. Quartile cut-points for serum liver markers in the NHANES 2011–2014 analytic sample.

Marker	Q1	Q2	Q3	Q4
GGT(u/L)	< 14	14–20	21–30	≥ 31

AST(U/L)	< 19	19–22	23–27	≥ 28
ALT(U/L)	< 16	16–20	21–27	≥ 28
ALP(U/L)	< 60	60–72	73–88	≥ 89
TP(g/L)	< 6.0	60–7.1	7.2–8.0	≥ 8.0

Note: Data are from the National Health and Nutrition Examination Survey (NHANES) 2011–2014, including 3,181 participants aged ≥60 years. Q1, Q2, Q3, and Q4 represent the first (lowest) to fourth (highest) quartiles, respectively. These cut-points were used to categorize serum liver markers into quartiles for the primary multivariable logistic regression analyses, with Q1 as the reference category. Abbreviations: GGT, gamma-glutamyl transferase; AST, aspartate aminotransferase; ALT, alanine aminotransferase; ALP, alkaline phosphatase; TP, total protein.

Table S5. Definition of covariates across models.

Model	Covariates
Model 1	Unadjusted
Model 2	Age, sex, education, marital
Model 3(primary)	Model 2 + BMI, alcohol, HDL, sleep duration
Model 4	Model 3 + race/ethnicity, PIR, smoking, diabetes, hypertension, CVD
Model 5	Model 4 + PHQ-9, eGFR, physical activity
Model 6	Model 5 + anticonvulsant use

Data are from the National Health and Nutrition Examination Survey (NHANES) 2011–2014, including 3,181 participants aged ≥60 years. All models accounted for the NHANES complex survey design using 4-year MEC examination weights (WTMEC2YR/2), strata (SDMVSTRA), and primary sampling units (SDMVPSU). Other liver markers (AST, ALT, ALP, TP) and LDL cholesterol were not included in the primary model; their influence was evaluated in sensitivity analyses as described in the Methods. Abbreviations: BMI, body mass index; HDL, high-density lipoprotein; PIR, poverty-income ratio; CVD, cardiovascular disease; PHQ-9, Patient Health Questionnaire-9; eGFR, estimated glomerular filtration rate.

Table S6 Effects of age and gender on learning and probe trial performance in the MWM

Effect	The learning task of MWM	The probe trial of MWM			
	AUC of escape	Escape latency	Number of times that the mice	Swimming time in the target	Swimming distance in the

	latency		crossed the removed platform	quadrant	Target quadrant
	p value	*p value*	*p value*	*p value*	*p value*
Strain	**5.98e-22**	**0.000451**	**0.000818**	**0.000991**	**0.000124**
Gender	0.697	0.628	0.499	0.498	0.609
Strain×Gender	0.089	0.680	0.672	0.915	0.609

Note: Data shown are *p values*. $p < 0.05$ signifies a significant influence of the factor on the respective indicator. AUC: area under the curve.

Table S7 Individual histopathological scoring of liver tissue from wild-type (WT) and 5xFAD mice across different ages

Mouse ID	Month	Hepatocellular ballooning score	inflammatory cell infiltration score	Necrosis score	Fibrosis score
WT-1	4.9	2	0	0	0
WT-2	4.9	2	0	0	0
WT-3	4.9	2	0	0	0
5xFAD-1	4.9	2	0	0	0
5xFAD-2	4.9	3	0	0	0
5xFAD-3	4.9	3	0	0	0
WT-1	14.9	3	0	0	0
WT-2	14.9	3	0	0	0
WT-3	14.9	3	0	0	0
5xFAD-1	14.9	3	0	0	0
5xFAD-2	14.9	3	0	0	0
5xFAD-3	14.9	3	0	0	0
WT-1	16.5	3	1	0	0
WT-2	16.5	3	0	0	0
WT-3	16.5	4	0	0	0
5xFAD-1	16.5	4	1	1	0
5xFAD-2	16.5	4	1	1	0
5xFAD-3	16.5	4	1	1	0
WT-1	19.5	4	1	1	0
WT-2	19.5	4	1	1	1
WT-3	19.5	3	2	1	0
5xFAD-1	19.5	4	2	1	1
5xFAD-2	19.5	4	1	1	1
5xFAD-3	19.5	4	1	1	1

Semi-quantitative grading of hepatocyte ballooning, inflammatory cell infiltration, necrosis, and fibrosis in liver sections from WT and 5xFAD mice at 4.9, 14.9, 16.5, and 19.5 months of age (n = 3 per strain per age group). Grading was performed according to the INHAND guidelines using a 0–4 scale, where 0 = within normal limits,

1 = very slight, 2 = slight, 3 = moderate, and 4 = severe. Detailed grading criteria are provided in Table S2 (semi-quantitative grading scale).

Table S8 Statistical analysis of liver function-related serum biochemical parameters.

	AST	ALT	ALP	GGT	TP
	P value	*P value*	*P value*	*P value*	*P value*
Age	0.070	0.256	0.349	0.067	0.214
Strain	0.206	0.277	**0.007**	**0.010**	**0.005**
Gender	0.368	0.900	0.171	0.625	0.063
Age×Gender	0.128	0.342	0.247	0.258	0.452
Age×Strain	0.484	0.289	0.435	0.332	0.053
Strain×Gender	0.263	0.777	0.696	0.079	0.757
Age×Gender×Strain	0.426	0.254	0.312	0.114	0.524

Note：*P-values* from the multifactorial analysis of variance were used to analyze the main effects of age in months, strain (WT and 5xFAD), and sex, as well as the interaction effects of strain × gender, age × gender, age × strain, and age × gender × strain. Blood biochemical parameters were measured using a fully automatic biochemical analyzer (BS-360S). Statistically significant values ($p < 0.05$) are shown in bold.

Table S9 Demographic and clinical characteristics of NHANES participants by memory status.

	level	Overall	LCP	NC
n		3181	417	2764
Age (median)		68.0[62.0,75.0]	70.0[64.0,77.0]	68.0 [62.0, 75.0]
Gender (%)	Female	1643 (51.7)	169 (40.5)	1474 (53.3)
	Male	1538 (48.3)	248 (59.5)	1290 (46.7)
Education (%)	Never attended school	397 (12.5)	81 (19.4)	316 (11.4)
	Elementary school	689 (21.7)	112 (26.9)	577 (20.9)
	Middle school	858 (27.0)	105 (25.2)	753 (27.2)
	High school	742 (23.3)	78 (18.7)	664 (24.0)
	College or above	495 (15.6)	41 (9.8)	454 (16.4)
Marital (%)	Divorced	306 (9.6)	57 (13.7)	249 (9.0)

	Married	1789 (56.2)	188 (45.1)	1601 (57.9)
	Never married	263 (8.3)	36 (8.6)	227 (8.2)
	Partner	182 (5.7)	26 (6.2)	156 (5.6)
	Separated	149 (4.7)	28 (6.7)	121 (4.4)
	Widowed	492 (15.5)	82 (19.7)	410 (14.8)
Alcohol consumption (%)	Mild	1582 (49.7)	180 (43.2)	1402 (50.8)
	Heavy	178 (5.6)	20 (4.8)	158 (5.7)
	None	1421 (44.7)	217 (52.0)	1204 (43.6)
BMI (%)	18.5-24.9	937 (29.5)	103 (24.7)	834 (30.2)
	<18.5	20 (0.6)	5 (1.2)	15 (0.5)
	25.0–29.9	2063 (64.9)	291 (69.8)	1772 (64.1)
	≥30.0	161 (5.1)	18 (4.3)	143 (5.2)
HDL (median)		50.0 [40.0, 60.0]	48.0 [38.0, 58.0]	51.0 [41.0, 61.0]
AST (median)		22.0 [18.0, 27.0]	22.0 [18.0, 27.0]	23.0 [19.0, 29.0]
ALT (median)		20.0 [15.0, 28.0]	20.0 [15.0, 28.0]	21.0 [16.0, 29.0]
ALP (median)		69.0 [57.0, 84.0]	69.0 [56.0, 83.0]	73.0 [60.0, 89.0]
GGT (median)		21.0 [14.0, 31.0]	21.0 [14.0, 30.0]	23.0 [17.0, 37.0]
TP (median)		7.1 [6.8, 7.4]	7.1 [6.8, 7.4]	7.0 [6.7, 7.3]
Sleep hours (median)		6.5 [5.5, 7.5]	6.0 [5.0, 7.0]	6.5 [5.5, 7.5]

Note: Data are presented as weighted median (IQR) for continuous variables and weighted percentage for categorical variables. Comparisons between LCP and NC groups were performed using survey-weighted t-tests for continuous variables and Rao-Scott chi-square tests for categorical variables, incorporating 4-year MEC examination weights (WTMEC2YR/2), strata (SDMVSTRA), and primary sampling units (SDMVPSU). LCP, low cognitive performance; NC, normal cognition.

Table S10 Sensitivity analysis of the association between serum GGT quartiles and LCP in the morning fasting subsample

Group	Characteristic	Range	n	OR	95% CI	*p-value*
Model1	GGT4					
	Q1	< 14	461	—	—	
	Q2	14–20	349	0.74	0.59, 0.94	0.012
	Q3	21–30	305	0.61	0.49, 0.77	<0.001
	Q4	≥ 31	164	0.52	0.42, 0.65	<0.001
Model2	GGT4					
	Q1	< 14	461	—	—	
	Q2	14–20	349	0.87	0.69, 1.10	0.257
	Q3	21–30	305	0.72	0.57, 0.91	0.007
	Q4	≥ 31	164	0.63	0.50, 0.79	<0.001
Model3	GGT4					
	Q1	<14	461	—	—	
	Q2	14–20	349	0.88	0.69, 1.12	0.288
	Q3	21–30	305	0.73	0.57, 0.92	0.009
	Q4	≥ 31	164	0.63	0.50, 0.80	<0.001

This sensitivity analysis was restricted to participants in the morning fasting subsample (n=1279). Fasting subsample weights (WTSAF2YR) were used, and LDL was added to the fully adjusted model. Model 1: unadjusted. Model 2: adjusted for demographic factors (age, sex, educational, marital). Model 3: further adjusted for BMI, alcohol consumption, HDL, LDL, sleep duration, and other liver function markers. The first quartile (Q1) was used as the reference group. Results should be interpreted in the context of the reduced precision of this sensitivity analysis.

Table S11 Expanded-adjustment sensitivity analyses

Group	Characteristic	Range	n	OR	95% CI	*p-value*
Model3	GGT4					
	Q1	< 14	902	—	—	
	Q2	14–20	835	0.85	0.67, 1.08	0.184
	Q3	21–30	764	0.69	0.54, 0.88	**0.003**
	Q4	≥ 31	680	0.54	0.42, 0.70	**<0.001**
Model4	GGT4					
	Q1	< 14	902	—	—	—
	Q2	14–20	835	0.85	0.67, 1.10	0.207
	Q3	21–30	764	0.71	0.55, 0.91	0.008
	Q4	≥ 31	680	0.55	0.43, 0.73	<0.001
Model5	GGT4					
	Q1	<14	902	—	—	—
	Q2	14–20	835	0.87	0.67, 1.12	0.266
	Q3	21–30	764	0.71	0.56, 0.95	0.018
	Q4	≥ 31	680	0.58	0.44, 0.77	<0.001

Model6	GGT4					
	Q1	<14	902	—	—	—
	Q2	14–20	835	0.88	0.68, 1.14	0.327
	Q3	21–30	764	0.74	0.56, 0.97	0.030
	Q4	≥ 31	680	0.59	0.45, 0.79	<0.001

Model 3: adjusted for age, sex, education, marital status, BMI, alcohol consumption, HDL, sleep duration, and other liver biochemical markers. Model 4: Model 3 plus race/ethnicity, poverty-income ratio, smoking status, diabetes, hypertension, and cardiovascular disease. Model 5: Model 4 plus PHQ-9 score, eGFR, and physical activity. Model 6: Model 5 plus anticonvulsant medication use. Q1 was the reference category. Survey-weighted logistic regression accounting for NHANES strata, primary sampling units, and examination weights.

Table S12. Collinearity diagnostics for the multivariable model.

	GVIF	Df	GVIF^(1/(2*Df))
GGT	1.02	1	1.01
Age	1.52	1	1.23
Gender	1.15	1	1.07
Education	1.33	4	1.04
Marital	1.64	5	1.05
Race	1.45	4	1.05
Sleep hours	1.02	1	1.01
BMI	1.13	2	1.03
HDL	1.20	1	1.09

Data are from the National Health and Nutrition Examination Survey (NHANES) 2011–2014, including 3,181 participants aged ≥60 years. Collinearity was assessed using generalized variance inflation factors (GVIFs). Commonly used thresholds are GVIF^(1/(2×Df)) > 2.24 (corresponding to VIF > 5) for moderate collinearity and > 3.16 (corresponding to VIF > 10) for severe collinearity. In this model, all GVIF^(1/(2×Df)) values were below 1.23, indicating no substantial multicollinearity. Abbreviations: GVIF, generalized variance inflation factor; Df, degrees of freedom; GGT, gamma-glutamyl transferase; BMI, body mass index; HDL, high-density lipoprotein.

Table S13 Multivariate logistic regression analysis of the association between ALP and LCP in the population

Group	Characteristic	OR	95% CI	*p-value*
Model1	ALP4			
	Q1	—	—	
	Q2	0.84	0.67, 1.06	0.138
	Q3	0.71	0.57, 0.89	**0.003**
	Q4	0.59	0.47, 0.73	**<0.001**
Model2	ALP4			
	Q1	—	—	
	Q2	0.98	0.77, 1.24	0.862
	Q3	0.89	0.70, 1.11	0.301
	Q4	0.84	0.67, 1.05	0.123
Model3	ALP4			

	Q1	—	—	
	Q2	1.01	0.80, 1.28	0.946
	Q3	0.94	0.74, 1.18	0.582
	Q4	0.93	0.74, 1.17	0.533

Note: Model 1: Unadjusted. Model 2: Adjusted for demographic factors (age, gender, education, marital). Model 3 (primary): Further adjusted for BMI, alcohol consumption, HDL, and sleep duration. Other liver markers (AST, ALT, ALP, TP) and LDL were not included in the primary model. ALP4 represents ALP levels categorized into quartiles (Q1-Q4), with Q1 as the reference group. OR, Odds Ratio; CI, Confidence Interval**.** $p < 0.05$ indicates a significant correlation

Table S14 Multivariate logistic regression analysis of the association between ALT and LCP in the population

Group	Characteristic	OR	95% CI	*p-value*
Model1	ALT4			
	Q1	—	—	
	Q2	1.0	0.81, 1.22	0.961
	Q3	1.09	0.88, 1.35	0.414
	Q4	1.15	0.92, 1.42	0.219
Model2	ALT4			
	Q1	—	—	
	Q2	1.00	0.81, 1.24	0.972
	Q3	1.09	0.87, 1.36	0.456
	Q4	1.03	0.81, 1.30	0.815
Model3	ALT4			
	Q1	—	—	
	Q2	1.02	0.82, 1.26	0.881
	Q3	1.13	0.89, 1.42	0.310
	Q4	1.15	0.88, 1.52	0.307

Note: Model 1: Unadjusted. Model 2: Adjusted for demographic factors (age, gender, education, marital). Model 3 (primary): Further adjusted for BMI, alcohol consumption, HDL, and sleep duration. Other liver markers (AST, ALT, ALP, TP) and LDL were not included in the primary model. ALT4 represents ALT levels categorized into quartiles (Q1-Q4), with Q1 as the reference group. OR, Odds Ratio; CI, Confidence Interval**.** $p < 0.05$ indicates a significant correlation

Table S15 Multivariate logistic regression analysis of the association between AST and LCP in the population

Group	Characteristic	OR	95% CI	*p-value*
Model1	AST4			
	Q1	—	—	
	Q2	1.02	0.83, 1.25	0.881
	Q3	1.11	0.89, 1.39	0.343
	Q4	0.94	0.76, 1.17	0.586

Model2	AST4			
	Q1	—	—	
	Q2	1.06	0.85, 1.31	0.620
	Q3	1.22	0.97, 1.53	0.095
	Q4	1.04	0.83, 1.30	0.734
Model3	AST4			
	Q1	—	—	
	Q2	1.01	0.81, 1.26	0.917
	Q3	1.12	0.88, 1.43	0.354
	Q4	0.93	0.70, 1.24	0.622

Note: Model 1: Unadjusted. Model 2: Adjusted for demographic factors (age, gender, education, marital). Model 3 (primary): Further adjusted for BMI, alcohol consumption, HDL, and sleep duration. Other liver markers (AST, ALT, ALP, TP) and LDL were not included in the primary model. AST4 represents AST levels categorized into quartiles (Q1-Q4), with Q1 as the reference group. OR, Odds Ratio; CI, Confidence Interval. $p < 0.05$ indicates a significant correlation

Table S16 Multivariate logistic regression analysis of the association between TP and LCP in the population

Group	Characteristic	OR	95% CI	*p-value*
Model1	TP4			
	Q1	—	—	
	Q2	0.91	0.75, 1.12	0.390
	Q3	1.21	0.97, 1.51	0.095
	Q4	1.00	0.81, 1.24	0.968
Model2	TP4			
	Q1	—	—	
	Q2	0.86	0.70, 1.07	0.170
	Q3	1.13	0.90, 1.43	0.300
	Q4	0.94	0.75, 1.18	0.597
Model3	TP4			
	Q1	—	—	
	Q2	1.02	0.81, 1.29	0.534
	Q3	0.98	0.78, 1.23	0.346
	Q4	0.97	0.77, 1.21	0.362

Note: Model 1: Unadjusted. Model 2: Adjusted for demographic factors (age, gender, education, marital). Model 3 (primary): Further adjusted for BMI, alcohol consumption, HDL, and sleep duration. Other liver markers (AST, ALT, ALP, TP) and LDL were not included in the primary model. TP4 represents TP levels categorized into quartiles (Q1-Q4), with Q1 as the reference group. OR, Odds Ratio; CI, Confidence Interval. $p < 0.05$ indicates a significant correlation

Table S17 Nonlinear association between serum GGT and LCP across models.

Model	P for nonlinearity	Approximate inflection point	Overall interpretation
Model 3	<0.001	19.4 U/L	Significant nonlinear association
Model 4	0.006	19.8 U/L	Pattern broadly preserved
Model 5	0.002	20.1 U/L	Pattern broadly preserved
Model 6	0.004	20.0 U/L	Pattern broadly preserved

Data are from the National Health and Nutrition Examination Survey (NHANES) 2011–2014, including 3,181 participants aged ≥60 years. Nonlinearity was assessed using restricted cubic splines (RCS) with three knots. Variables adjusted by the model correspond to Table S5

Table S18 Subgroup analysis of the association between serum GGT and LCP.

Variable	N	OR (95% CI)	*p value*	*p* for interaction
Overall	3181			
GGT 10-60	2481	1 [Reference]		
GGT⩾60	318	0.67 (0.52-0.85)	0.001**	
GGT < 10	382	1.84 (1.23-2.76)	0.003**	
age				0.065
>65	1909			
GGT 10-60	1489	1 [Reference]		
GGT⩾60	229	0.70 (0.43-1.14)	0.155	
GGT < 10	191	0.90 (0.47-1.71)	0.747	
<=65	1272			
GGT 10-60	992	1 [Reference]		
GGT⩾60	89	0.62 (0.47-0.82)	0.001**	
GGT < 10	191	2.41 (1.40-4.15)	0.001**	
gender				0.243
Female	1643			

GGT 10-60	1282	1 [Reference]		
GGT⩾60	115	0.74 (0.53-1.03)	0.072	
GGT < 10	246	1.10 (0.47-2.58)	0.82	
Male	1538			
GGT 10-60	1200	1 [Reference]		
GGT⩾60	200	0.57 (0.39-0.81)	0.002**	
GGT < 10	138	2.12 (1.33-3.37)	0.001**	
education				0.564
Never attended school	397			
GGT 10-60	298	1 [Reference]		
GGT⩾60	39	1.29 (0.61-2.72)	0.511	
GGT < 10	60	1.22 (0.34-4.36)	0.757	
Elementary school	689			
GGT 10-60	537	1 [Reference]		
GGT⩾60	69	0.70 (0.41-1.17)	0.174	
GGT < 10	83	1.16 (0.48-2.78)	0.738	
Middle school	858			
GGT 10-60	669	1 [Reference]		
GGT⩾60	86	0.78 (0.47-1.29)	0.337	
GGT < 10	103	1.73 (0.83-3.61)	0.146	
High school	742			
GGT 10-60	586	1 [Reference]		
GGT⩾60	74	0.54 (0.35-0.83)	0.005**	
GGT < 10	82	1.76 (0.81-3.82)	0.156	
College or above	495			
GGT 10-60	396	1 [Reference]		

GGT≥60	49	0.50 (0.23-1.07)	0.073	
GGT < 10	50	2.79 (0.67-11.55)	0.156	
Marital				0.731
Divorced	306			
GGT 10-60	239	1 [Reference]		
GGT≥60	30	0.71 (0.39-1.30)	0.27	
GGT < 10	37	2.46 (0.58-10.41)	0.222	
Married	1789			
GGT 10-60	1395	1 [Reference]		
GGT≥60	179	0.68 (0.47-1.00)	0.049*	
GGT < 10	215	2.34 (1.19-4.62)	0.014*	
Never married	263			
GGT 10-60	197	1 [Reference]		
GGT≥60	27	0.49 (0.27-0.92)	0.027*	
GGT < 10	39	1.47 (0.58-3.70)	0.416	
Partner	182			
GGT 10-60	142	1 [Reference]		
GGT≥60	18	0.72 (0.29-1.77)	0.472	
GGT < 10	22	4.13 (0.56-30.62)	0.165	
Separated	149			
GGT 10-60	116	1 [Reference]		
GGT≥60	15	1.17 (0.42-3.25)	0.76	
GGT < 10	18	2.03 (0.25-16.48)	0.508	
Widowed	492			
GGT 10-60	369	1 [Reference]		
GGT≥60	49	0.76 (0.39-1.51)	0.435	

GGT < 10	74	0.86 (0.38-1.94)	0.724	
Alcohol consumption				0.168
Mild	1487			
GGT 10-60	1190	1 [Reference]		
GGT⩾60	148	0.60 (0.41-0.88)	0.008**	
GGT < 10	149	2.48 (1.30-4.71)	0.006**	
Heavy	273			
GGT 10-60	205	1 [Reference]		
GGT⩾60	54	0.43 (0.26-0.71)	0.001**	
GGT < 10	14	1.48 (0.19-11.31)	0.703	
None	1421			
GGT 10-60	1066	1 [Reference]		
GGT⩾60	142	0.89 (0.57-1.38)	0.596	
GGT < 10	213	1.43 (0.83-2.48)	0.201	
BMI				0.726
18.5-24.9	937			
GGT 10-60	731	1 [Reference]		
GGT⩾60	94	0.48 (0.28-0.83)	0.009**	
GGT < 10	112	1.59 (0.79-3.18)	0.194	
25.0-29.9	2063			
GGT 10-60	1625	1 [Reference]		
GGT⩾60	208	0.73 (0.55-0.96)	0.023*	
GGT < 10	250	1.91 (1.13-3.25)	0.016*	
<18.5	20			
GGT 10-60	16	1 [Reference]		
GGT⩾60	2	1.02 (0.12-9.00)	0.984	

GGT < 10	2	1.39 (0.29-6.66)	0.682	
≥30.0	161			
GGT 10-60	113	1 [Reference]		
GGT≥60	16	0.88 (0.36–2.15)	0.78	
GGT < 10	32	1.52 (0.55–4.20)	0.42	
Sleep duration				0.192
>=6	2386			
GGT 10-60	1861	1 [Reference]		
GGT≥60	239	0.60 (0.46-0.79)	0.003**	
GGT < 10	286	1.85 (1.18-2.89)	0.007**	
<6	795			
GGT 10-60	620	1 [Reference]		
GGT≥60	80	1.02 (0.59-1.79)	0.934	
GGT < 10	95	1.42 (0.55-3.66)	0.468	

GGT, gamma-glutamyl transferase; OR, odds ratio; CI, confidence interval. GGT was categorized using clinically meaningful cut-points: low (<10 U/L), normal (10–60 U/L, reference), and high (≥60 U/L). The upper cut-point of 60 U/L corresponds to the IFCC consensus upper reference limit for men and has been used in previous prospective cohort studies. The lower cut-point of 10 U/L is close to the lower limit of the reference interval reported in NHANES. These cut-points were used in subgroup analyses to facilitate interpretation of extreme values and comparison with clinical thresholds. n refers to the unweighted number of participants in each stratum. p for interaction was derived from product terms in the regression models. *p < 0.05; **p < 0.01.

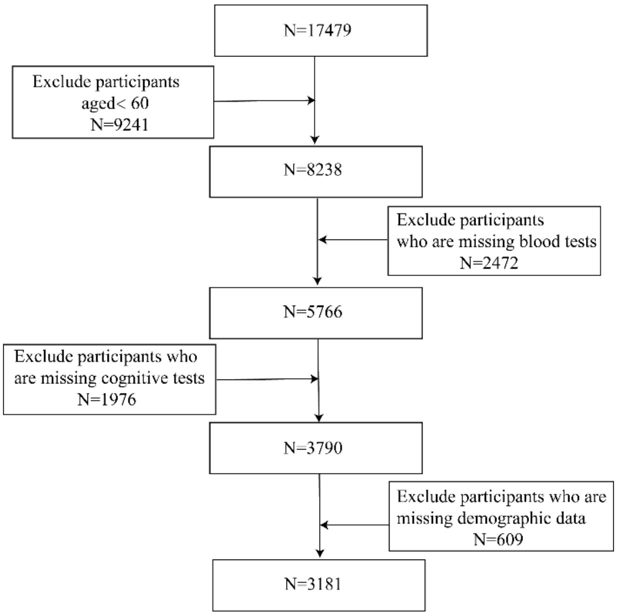

Figure S1 Flowchart of participant selection from the NHANES cohort.

A total of 17,479 individuals from NHANES (2011–2014) were initially considered. Finally, 3,181 eligible participants were included in the analysis.

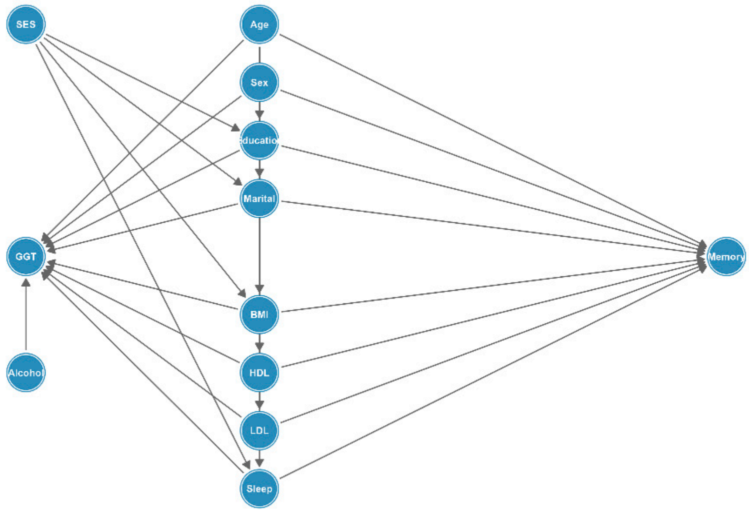

Figure S2. Directed acyclic graph (DAG) of the relationship between serum GGT and LCP.

Abbreviations: GGT, gamma-glutamyl transferase; BMI, body mass index; HDL, high-density lipoprotein; LDL, low-density lipoprotein; SES, socioeconomic status. The variables in the blue box are included as covariates in the final model.

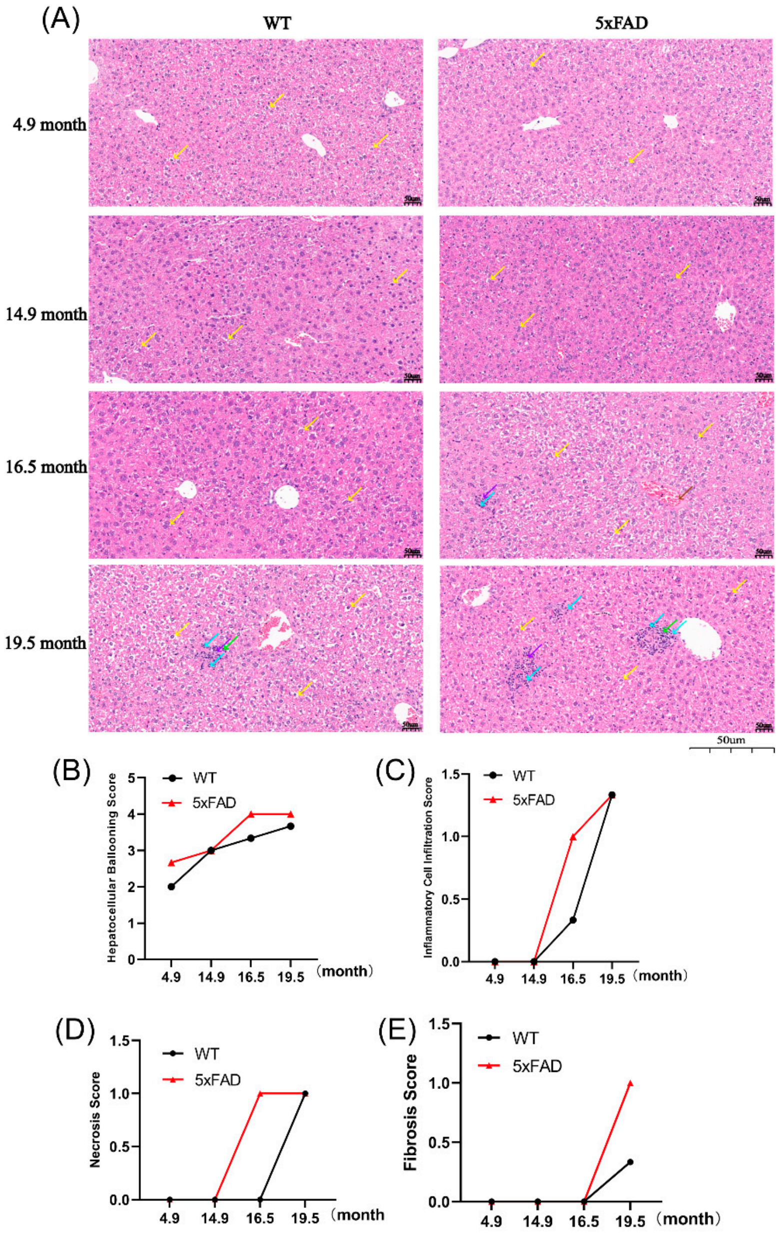

Figure S3 Age-related hepatic histopathology in 5xFAD and WT mice.

(A)Liver sections from WT (left) and 5xFAD (right) mice, aged 4.9, 14.9, 16.5, and 19.5 months. Images were taken at 20× magnification. Pathology grading (0–4) was performed according to INHAND guidelines (Table S2). Detailed semi-quantitative data corresponding to the pathology grades can be found in Table S7. (B) Grading of hepatocyte edema. (C)Grading of inflammatory cell infiltration in the liver tissue. (D) Grading of hepatocyte necrosis. (E)Grading of connective tissue proliferation (fibrosis) in the liver tissue. According to the International Harmonization of Nomenclature and Diagnostic Criteria (INHAND) guidelines, histopathological changes in mouse liver were graded as: 0, within normal limits; 1, very minimal; 2, minimal; 3, moderate; 4, marked. Take the average pathological score of mice in each age group.

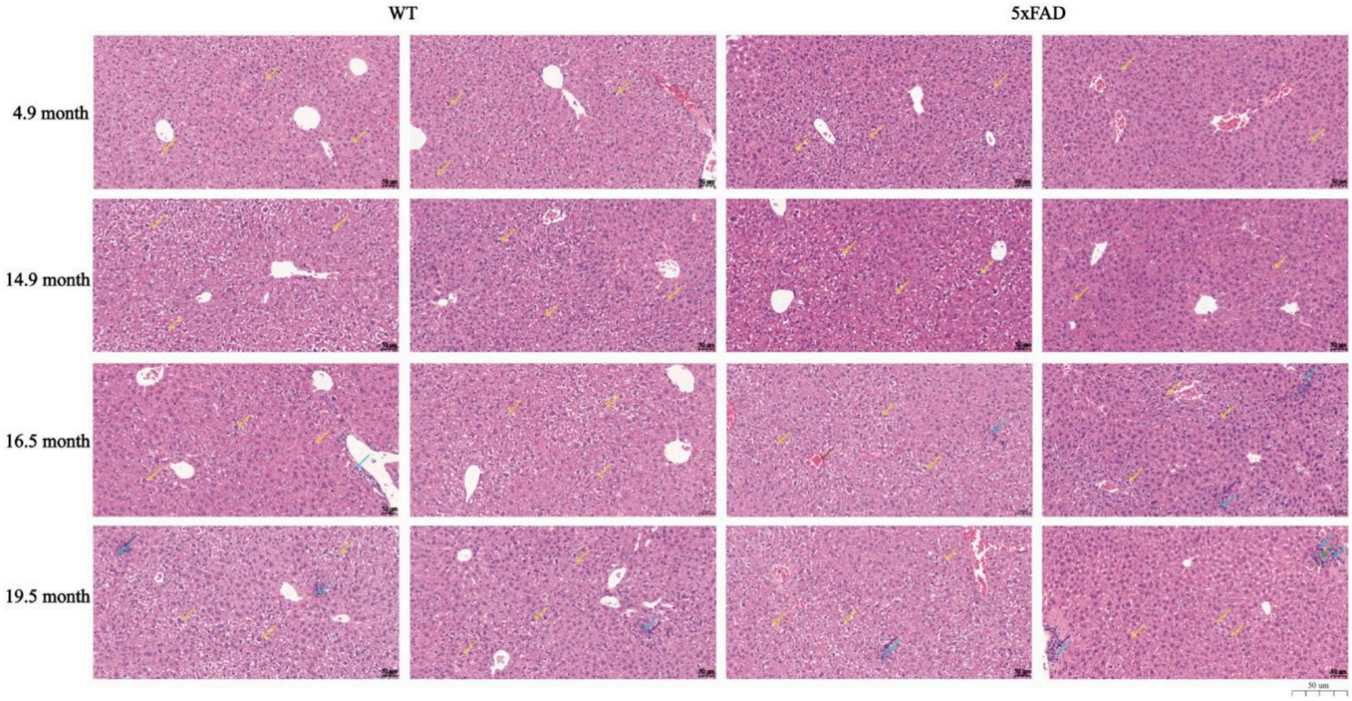

Figure S4 Remaining H&E-stained liver sections from 5xFAD and WT mice of different ages.

Liver sections from WT (left two columns) and 5xFAD (right two columns) mice at 4.9, 14.9, 16.5, and 19.5 months of age. Images were captured at 20× magnification. Pathological grading (0 - 4) was performed according to INHAND guidelines (Table S2). Detailed semi-quantitative data corresponding to the pathological grades can be seen in Table S7.
